# Hepatitis C Virus, Cholesterol and Lipoproteins — Impact for the Viral Life Cycle and Pathogenesis of Liver Disease

**DOI:** 10.3390/v5051292

**Published:** 2013-05-22

**Authors:** Daniel J. Felmlee, Mohamed Lamine Hafirassou, Mathieu Lefevre, Thomas F. Baumert, Catherine Schuster

**Affiliations:** 1 Inserm, U1110, Institute of Virology, Strasbourg 67000, France; E-Mails: felmlee@unistra.fr (D.J.F.); mohamed-lamine.hafirassou@etu.unistra.fr (M.L.H.); mathieu.lefevre@etu.unistra.fr (M.L.); 2 Université de Strasbourg, Strasbourg 67000, France; 3 Pôle Hépato-digestif, Hôpitaux Universitaires de Strasbourg, Strasbourg 67000, France

**Keywords:** Hepatitis C virus, lipoproteins, apolipoproteins, apoE, apoB, cholesterol, triglyceride, viral attachment, entry, assembly, secretion, viral immune escape, lipid disorder

## Abstract

Hepatitis C virus (HCV) is a leading cause of chronic liver disease, including chronic hepatitis, fibrosis, cirrhosis, and hepatocellular carcinoma. Hepatitis C infection associates with lipid and lipoprotein metabolism disorders such as hepatic steatosis, hypobetalipoproteinemia, and hypocholesterolemia. Furthermore, virus production is dependent on hepatic very-low-density lipoprotein (VLDL) assembly, and circulating virions are physically associated with lipoproteins in complexes termed lipoviral particles. Evidence has indicated several functional roles for the formation of these complexes, including co-opting of lipoprotein receptors for attachment and entry, concealing epitopes to facilitate immune escape, and hijacking host factors for HCV maturation and secretion. Here, we review the evidence surrounding pathogenesis of the hepatitis C infection regarding lipoprotein engagement, cholesterol and triglyceride regulation, and the molecular mechanisms underlying these effects.

## 1. Introduction

For the approximately 170 million individuals infected with hepatitis C virus (HCV), there is no vaccine available and the standard of care therapeutic options prior to inclusion of direct antiviral agents (DAAs) have been effective for only 50% of patients, for the most prevalent phylogenetically distinct group by 30% nucleotide sequence, genotype 1 [1]. This presents a major health problem, as HCV infection is a leading cause of chronic hepatitis, liver cirrhosis, and liver cancer worldwide. Chronic HCV infection is typified by slow progression to cirrhosis and advanced liver disease. Many individuals, who acquired the infection as young adults in the 1970s, are now presenting with serious liver disease, resulting in an increasing prevalence of hepatocellular carcinoma and cirrhosis over the past decade [2]. The recently developed DAAs are becoming a new arm for the standard treatment for HCV and promise to increase therapeutic efficacy significantly [1,3,4,5], but these options are still limited by the emergence of resistance mutations. Treatments to date are specifically aimed at genotype 1 HCV infection, leaving a large swath of patients without effective treatment. Furthermore, those patients who did not respond favorably to the current standard treatment have a decreased response rate to DAAs [6,7]. Therefore, while a new era of HCV treatment is on the horizon, the pathogenesis and disease resulting from HCV infection remain critical issues that need to be addressed.

**Figure 1:**
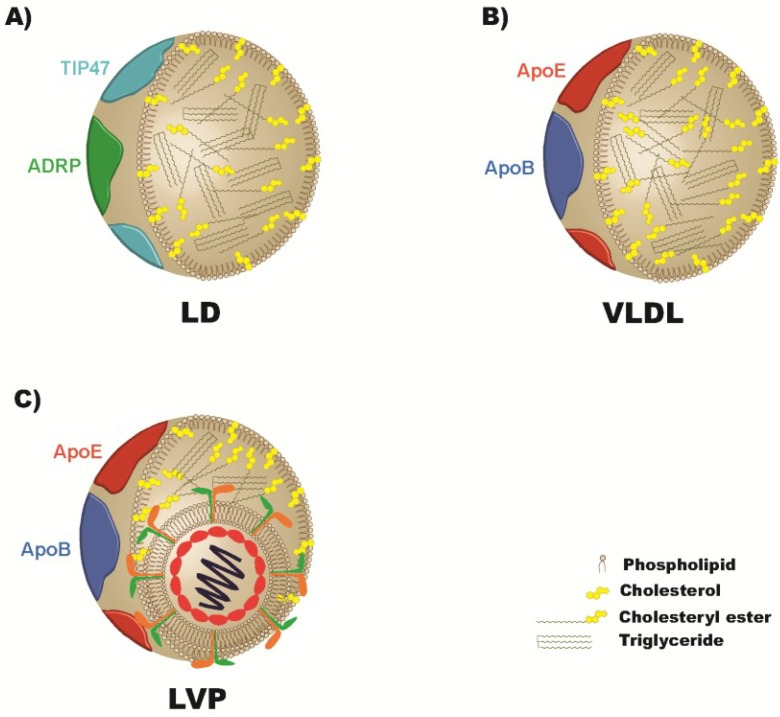
Functional comparison between cytosolic lipid droplets, very-low density lipoprotein (VLDL), and lipoviral particles (LVP). (**A**) Hepatic cytosolic lipid droplets (LD) and (**B**) VLDL share common properties and functions albeit in different compartments. The lipid components of both LDs and VLDL particles consists of a hydrophobic triglyceride and cholesteryl-ester core surrounded by a free cholesterol and phospholipid monolayer where amphipathic proteins may be embedded or peripherally associated, including adipose differentiation-related protein (ADRP/PLIN2) and Tail interacting Protein of 47 kDa (TIP47/PLIN3) for LDs [18,19], and apolipoproteins (apos) including apoB, apoE, apoCI-III for VLDL [20]. The functions of these proteins are analogous: to stabilize assembly, to provide docking sites for the appropriate receptors and regulatory proteins, and to regulate access to underlying lipids [21]. (**C**) Hepatitis C virus (HCV) particles in patient sera circulate in complexes with host lipoproteins as lipoviral particles, which are enriched in triglyceride, cholesterol, and several apolipoproteins [22,23].

Even before the isolation of HCV as the viral agent causing non-A non-B hepatitis, it was known that this pathologic agent had a unique interaction with lipids and lipoproteins. Most notably, the accumulation of neutral lipids in cytosolic lipid droplets in hepatocytes was defined as a pathologic hallmark of hepatitis C virus infection [8]. Early biophysical characterizations of virus particles in patient serum also revealed the virus to be highly heterogenous in buoyant density due to association with host lipoproteins [9,10]. The lipoprotein association of the virus particle was examined further both from human serum and from particles developed from hepatoma cells in cell culture (HCVcc). These characterizations have revealed virus particles that have both aspects of typical virions and aspects more similar to host-lipoproteins. These infectious hybrid particle complexes have been termed lipoviral particles (LVP) to highlight their complex nature (Figure 1). Since the HCV virion utilizes lipoproteins in such a unique way, lipoprotein metabolism research has illuminated understanding of the virus-host interactions of HCV. We outline here some of the most intriguing results of the role of cholesterol and other lipids in HCV pathogenesis, and describe their role in the steps of the HCV life cycle including entry, replication, and assembly.

## 2. Lipids, Apolipoproteins, and HCV Pathogenesis

Clinical evidence indicates that HCV infection is not only intimately linked to the metabolism of lipids within the hepatocytes that HCV infects, but dysregulates circulating lipoprotein metabolism as well. The liver is the central organ of lipid homeostasis for the entire body, through production and uptake of lipoproteins. Triglycerides (TG) are packaged in lipoproteins surrounded by a phospholipid, cholesterol, and amphipathic protein monolayer to deliver lipids produced or absorbed from the liver and intestine respectively to other organs (Figure 1). While HCV hijacks elements of the very-low density lipoprotein (VLDL) secretion pathway for the production of infectious particles, the LVP that circulate in an infected individual indicate that HCV virions are not only associated with hepatically derived triglyceride-rich lipoproteins (TRL) containing apoB-100, but are also associated with intestinally derived lipoproteins containing apoB-48 [11,12], an isoform of apoB exclusively generated from the small intestine. The presence of this subpopulation of LVP indicates an active exchange of virions between lipoproteins [12]. Aside from infectious LVP, HCV envelope proteins have been detected on the surface of lipoproteins devoid of infectious nucleocapsids, in so called empty LVP (eLVP), possibly contributing to the physiopathology of the disease [13]. The functional advantage of the association of virions with host lipoproteins has not been completely elucidated, though evidence suggests utilization of lipoprotein components may both mediate attachment to lipoprotein receptors, and obscure circulating viral particles from immunoglobulin recognition, thereby allowing the virus to escape immune surveillance [14,15]. It must be determined whether these mechanisms are clear priorities, given the implications in vaccine design and the possibility that abrogating these mechanisms may have important clinical significance in preventing infection post-liver transplantation [16,17].

Aside from the physical association of HCV components with lipoproteins, intracellular lipids play key roles throughout the HCV life cycle. HCV replication requires numerous factors involved in lipid metabolism and HCV assembly and production depends on elements of VLDL assembly. Chronic HCV infection is associated with deregulated lipid homeostasis favoring triglyceride accumulation in the liver [24,25]. HCV infection may contribute to this accumulation through transcriptional activation of lipogenic genes favoring lipid synthesis in patients [26,27]. HCV infected chimpanzees likewise revealed transcriptional induction of these genes via activation of sterol response element binding proteins (SREBPs), important transcription factors involved in cholesterol and fatty acid regulation [28]. The crucial role of the viral proteins core, NS2, and NS4B, in activating of SREBP and the retinoid X receptor (RXR) alpha pathways was further confirmed both in transgenic mouse models [29,30], and in cell culture systems [31,32,33]. However, clinical studies have indicated that HCV induced overexpression of lipogenic genes may exert a strong influence on inflammation and fibrosis progression of the infected liver, rather than causing the lipid accumulation observed in hepatic steatosis [34]. Sophisticated stable-label studies indicate that patients with HCV infection have increased fatty acid synthesis and diminished cholesterol synthesis compared to uninfected individuals [35]. While the studies are limited by patient numbers, stable-label studies may further illuminate HCV-associated metabolic disturbances in future studies.

In HCV infected patients, the prevalence of steatosis is twofold higher than in HBV infected patients [36,37], demonstrating a clear correlation between HCV infection and non-alcoholic fatty liver (NAFL) disease. This correlation is particularly strong in genotype 3 infection relative to other genotypes, suggesting genetic variation of HCV contributes to lipid accumulation. Further evidence of a direct effect of the virus is the fact that the steatotic grade correlates with the HCV RNA quantity present both in the liver [24], and in the serum of infected patients [38,39]. Those patients with HCV infection who manifest steatosis, are also more likely to present hypobetalipoproteinemia (diminished serum levels of apoB bearing lipoproteins) and diminished serum cholesterol levels [25,36,40,41]. These observations indicate that HCV associated steatosis may be a sequela of diminished triglyceride export by modulated apolipoprotein B bearing lipoprotein production [25]. There is a correlation of HCV genotype 3 infection with both steatosis and hypocholesterolemia [25]. Recent metabolomic analysis also indicates that genotype 3 HCV infection inhibits cholesterol synthesis as there is a lack of late step intermediate metabolites in patients infected with genotype 3, but not genotype 2 HCV [42]. Indeed, the rate-limiting enzyme of VLDL production, microsomal triglyceride transfer protein (MTP), is transcriptionally repressed by HCV gene expression both *in vitro* [43], and *in vivo* [44], and associates with steatosis [44]. Recent studies suggest that the virus-induced dysregulation of apoB-100 secretion is mediated by increased ferritin heavy chain levels [45]. Indeed, an inverse correlation between ferritin and secreted apoB-100 concentrations is found both in JFH-1 HCVcc and HCV-infected patients, indicating a possible explanation for the onset of virus-induced liver steatosis [45]. Steatosis is not only caused by HCV, but is also linked to pathogenesis and enhanced disease progression. Chronic HCV infection is also strongly associated with insulin resistance, which might be a consequence of impaired insulin signaling and activation of inflammatory markers such as TNF-alpha and the suppressor of cytokine signaling (SOCS) family proteins. This in turn deregulates fatty acid synthesis and leads to hepatic steatosis. In parallel, the HCV core protein increases the activity of peroxisome proliferator-activating receptor (PPAR)–alpha and gamma in hepatocytes contributing to deregulation of fatty acid beta-oxydation and insulin sensitivity (for a review see [46]). Finally, Fujino *et al.* demonstrated that lipid metabolism genes like the SREBP family genes expression are modified through this mechanism [26].

The viral effect on patient serum lipid profile has recently been confirmed in a large scale study in China including 11,000 patients that reported HCV viremia statistically associating with lower serum cholesterol and TG levels [47]. This pathology can also be observed in transgenic mice expressing the HCV polyprotein [30]. Diminished triglyceride levels may also play an important role in the severity of the infection, since an Egyptian study highlighted that high circulating TG levels during acute infection associates with spontaneous clearance of HCV [41]. Moreover, the VLDL-TG to non-VLDL-TG ratio, a measure of the proportion of large TRLs, negatively associated with disease progression; patients with more advanced fibrosis were lacking in large TRLs [48].

Biochemical analysis of HCVcc using a purification scheme involving an epitope tagged envelope protein displayed apoE on the virion surface [49]. The extent of this association is dependent on growth conditions, since HCVcc grown in serum free media are less capable of immunoprecipitation with apoE antibodies than HCVcc grown in media supplemented with 10% fetal bovine serum [50]. Three isoforms of apoE are present in the human population, determined by cysteine residue substitutions at positions 112 and 158, termed E2, E3 and E4. These apoE isoforms affect lipoprotein uptake by hepatocytes due to differential affinity to LDL receptor (LDLr). Given the primary role of apoE in the HCV life cycle, several studies investigated the possibility of a correlation between apoE isoform and hepatitis C infection. Using the HCVcc system, it remains controversial, but apoE genotype seems to have little effect in altering infectivity [51,52,53,54]. Comparison of genotype and allele distribution with those of healthy controls yielded evidence of diminished progression of liver disease and viral clearance associated with the E2 allele, which may protect against establishment of chronicity via defective binding of LVP to the cellular receptors involved in HCV entry [55]. Similarly, apoE4, a contributing factor to both Alzheimer’s disease and cardiovascular disorders, appears to have a protective effect against HCV infection and slows fibrosis progression relative to other hepatotropic viral diseases [56,57]. Studies have suggested important roles in HCV infection played by single nucleotide polymorphisms of host genes involved in lipid metabolism, such as LDLr [58] and apoB-100, however these studies were limited by population size and conclusive results await the confirmation of larger-scale studies. 

As previously mentioned, HCV directly affects the composition of host circulating lipoproteins of HCV positive patient sera, for example through the generation of eLVP. These altered lipoproteins then in turn induce changes in the lipid metabolism of monocyte-derived macrophages [59]. Analysis of the apolipoprotein content of VLDL, LDL, and HDL fractions derived from sera of those with or without HCV infection showed a specific decrease in the apoA-I content in the LDL fraction [60]. This may represent a depletion of large HDL particles, providing clinical evidence that HCV and host lipoproteins are reciprocally influenced [60]. Similarly, Kim *et al.* observed an induction of apoC-IV transcription and increased hepatic triglyceride levels in hepatocytes of patients with chronic HCV infection [61]. An *in vitro* study confirmed aberrant transcriptional stimulation of apoC-IV in the presence of core, driven by the KU antigen and the PPAR gamma/RXRalpha complex [61].

Rowell *et al.* investigated these links clinically by measuring several lipid and lipoprotein parameters including total cholesterol, triglyceride, low-density lipoprotein cholesterol (LDL-C), high-density lipoprotein cholesterol (HDL-C), apoA-I, -B, -CIII, and -E in the sera of patients who both did or did not clear chronic HCV infection. Interestingly, multiple logistic regression analysis revealed diminished serum apoC-III levels (−25%) to be the factor that most strongly associated with both chronic infection and the increasing severity of hepatic fibrosis. The authors of this study propose that apoC-III levels may potentially act as a possible marker of HCV disease progression [62].

Several studies have indicated a link between the successful outcome of antiviral treatment and observed lipid metabolism parameters of the patient. Both hepatic steatosis and insulin resistance impair treatment response to interferon α (IFNα) [63,64,65]. Moreover, hypobetalipoproteinemia and diminished serum cholesterol levels are reversed after effective response to antiviral therapies, indicating the direct role of the virus on cholesterol and LDL levels [25,66,67,68]. However, the decreased cholesterol and LDL levels associated with HCV does not appear to translate into a cardioprotective role, as carotid atherosclerosis is increased in HCV patients, perhaps due to increased insulin resistance and metabolic syndrome [69]. Residues Arg70/Leu91 in the HCV core region, along with high LDL-C serum levels are associated with early and sustained virological response [70]. Furthermore, interferon sensitivity is characterized by low LVP ratios and low apoE levels along with higher LDL-C and IL28B rs12979860 CC in HCV genotype 1 infected patients [71], while null-response is associated with increased LVP ratio. Lipoprotein profiles more indicative of metabolic syndrome, such as a high triglyceride/HDL ratio; likewise, they were indicative of an increased LVP ratio for genotype 1 patients [72]. The association of lipids with peg-interferon treatment response suggests that lipid modulation may be an effective strategy to modify interferon sensitivity [71,73].

## 3. Treatments Targeting Lipid Metabolism

The close relationship between host lipids and the HCV life cycle generates opportunities of new therapeutic options that target lipid regulation. Indeed, host factor targeting overcomes viral resistance due to emerging escape variants and genotype variability. Several lipid modulating agents, which were initially tested for their cardioprotective roles, may increase the efficacy of antiviral therapies, such as insulin sensitizing drugs and statins. Since insulin resistance and steatosis can modify antiviral treatment outcome, and since IR and steatosis enhance progression of the disease, it has been proposed to combine standard of care treatment and administration of insulin sensitizer such as metformin [74] or thiazolidinedione to manage insulin resistance and in turn induce a SVR [75,76]. Statins are inhibitors of 3-hydroxy-3-methylglutaryl coenzyme A reductase (HMGCR), a limiting step of cholesterol synthesis, and are widely used to modulate cholesterol levels in patients at risk of heart disease, or with familial hypercholesterolemia. The combination of statins with standard of care treatment can decrease hepatic steatosis and improve standard of care treatment efficacy [77,78,79]. 

Despite promising effects observed in *in vitro* studies [80], the administration of statins alone lead to contradictory effects on HCV viral load [81,82,83,84]. However, the combination of statins both with IFNα alone, or with IFNα and ribavirin showed a strong synergistic effect [79,85,86,87]. Other strategies are under investigation in clinical trials, such as combination with PPAR agonists, antagonists, or nicotinic acid. Promising lipid modulating results in humanized mice and chimp models have indicated utility for apoB antisense miRNA (mipomersen) [88] or micro-RNA 122 (miR-122) antagonists [89] for their clinical utility as antivirals [90]. However, proof-of-concept studies for HCV therapy remains to be determined. Moreover, natural compounds that affect lipid metabolism such as the grapefruit flavonoid naringenin [91,92] as well as Epigallocatechin-3-gallate (EGCG), an anti-oxidant molecule isolated from the green tea show an antiviral effect. EGCG inhibits HCV entry at the viral attachment step [93,94], and EGCG and derivatives also inhibits viral replication via cyclooxygenase 2 and can decrease viral induced inflammation [95]. Green tea compounds as well as naringenin may act as interesting natural complement diet to lower the progression of HCV induced liver disease, however *in vivo* clinical trial data are yet not available.

## 4. Apolipoproteins and the Viral Particle

Viral particles purified from patient sera were observed for the first time by electromicroscopy in 1994 [96]. The particles observed were spherical and heterogeneous in size and density, corroborating previous findings [97,98,99], and confirmed later by several other groups [22,23,100]. Biochemical analysis using sucrose gradients of infected patients sera further showed that HCV RNA is distributed over a wide range of densities from 1.20 g/cm^3^ to 1.03 g/cm^3^ [101]. The fractions containing the highest amount of RNA are between 1.12 g/cm^3^ and 1.04 g/cm^3^ [23,102], though this distribution is highly dynamic and affected by lipoprotein metabolism [12]. Density heterogeneity is in part explained by association of the virus both with immunoglobulins [22,103] and different lipoprotein classes [9,10,22,23,97,102,103]. Associations with lipoproteins were further confirmed by immunoprecipitation of the HCV RNA containing fractions with anti-apolipoprotein antibodies against apoB [9,23] or apoE [22,23]. A major advance in HCV research occurred in 2005 with the discovery that the highly replicative HCV JFH1 strain, was capable of producing infectious virions from select hepatoma cell lines in culture [104,105,106]. Early studies of HCVcc revealed that the majority of HCV RNA produced is higher in density than the majority of infectious particles. Analysis of HCVcc largely confirmed previous observations obtained with human sera regarding density and size heterogeneity as well as the LVP nature of the HCVcc [107]. HCVcc permitted manipulation of HCV producing cells, and it quickly became apparent that altering VLDL components had dramatic effects on HCV particle production. Chemical inhibition of MTP dramatically inhibits HCVcc production, as well as genetic silencing of apolipoproteins apoE and apoB [108,109]. The average density of LVP from human sera is lower compared to HCVcc (1.05 g/cm^3^ *vs.* 1.10 g/cm^3^) [104,105,107]. This can be explained at least in part by the differences in the neutral lipid content of the LVP produced in cell culture *vs.* serum derived virions, since the cell type that sustains HCV replication, Huh7, secrete a majority of LDL-sized rather than VLDL-sized apoB bearing particles [110]. Density may also vary based on the cell type producing the virus [107,111,112,113,114]. Indeed, LVP produced in human liver engrafted mice [113], as well as in primary human hepatocytes [114], more closely resemble particles purified from patients infected with HCV. HCVcc passaged through different animal models or cell types revealed a higher specific infectivity of the lowest-density populations [104,105,107,113]. However, engineering of cells to produce a majority of VLDL does not radically change the virus distribution to low-density, indicating a possible genetic contribution for LVP formation [115]. Analysis of the lipid composition of the HCVcc particles revealed similar composition to LDL and VLDL, in cholesterol, cholesteryl esters, phospholipids, and sphingolipids [49,102,116]. The presence of apolipoproteins such as apoE, apoB, apoCI, apoA1 have also been reported to be HCVcc components through immunoprecipitation studies [11,22,60,108,109,117,118]. It is noteworthy that apolipoproteins play an important role in HCV entry, assembly, and export steps [49,60,108,109,118,119,120]. This is the consequence of VLDL remnant uptake, VLDL assembly, and export mechanisms being used by the virus. Indeed, it is assumed that HCV highjacks VLDL assembly by associating with nascent VLDL in the ER lumen to egress the infected cell [109]. Apolipoprotein content analysis of purified HCVcc showed that each particle bears approximately 300 molecules of apoE at its surface [49], a remarkable enrichment since the estimates of apoE molecules per VLDL are 5–7 [121]. Apart from apoE, apoC1 has been shown to associate with HCV particles derived from the sera of chimpanzees, patient derived LVP, and HCVcc [118]. Data indicates that apoCI may play an important role in HCV endosomal fusion immediately after HCV entry [122]. In contrast to LVPs, purified HCVcc were not immunoprecipitated with anti-apoB antibodies, indicating that HCVcc contain little or no apoB relative to patient-derived LVPs. This observation may explain the density shift observed between both LVPs and HCV from primary hepatocyte cultures compared to HCVcc [114]. In Caco-2 cells, an intestinal cell line which possesses intact VLDL assembly machinery, ectopic expression of E1 and E2 viral glycoproteins give raise to capsid-free apoB-bearing TRL that are similar to eLVPs observed in patients [117]. These data indicate the close association between HCV glycoproteins and host cell lipoproteins even in the absence of viral capsids, a finding recently confirmed in observations of E1/apoB interactions [123]. Furthermore, recent evidence suggests that basic residues of HCV E2 protein may be important for the infectivity of low-density particles [124].

## 5. Lipoprotein and Cholesterol’s Role in Viral Entry

**Figure 2:**
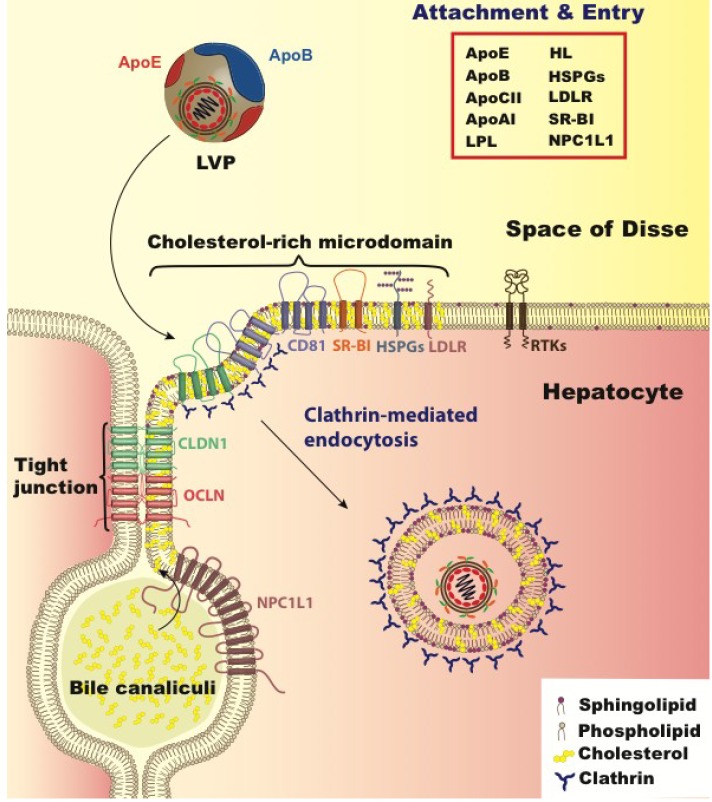
HCV hepatocyte entry. HCV entry into human hepatocytes is a multi-step process in which many host factors are involved including heparan sulfate proteoglycans (HSPGs), the low density lipoprotein receptor (LDLr), the scavenger receptor class B type I (SR-BI), tetraspanin CD81, the tight junction proteins, claudin-1 (CLDN1) and occludin (OCLN), receptor tyrosine kinases (RTKs),and the Niemann–Pick C1-like 1 (NPC1L1) (reviewed in [131]). The LVP initially binds HSPGs and LDLR via apoE. Subsequent interaction with SR-BI, CD81, CLDN1, and OCLN leads to viral internalization in cholesterol-rich microdomains via clathrin-mediated endocytosis. Other entry factors such as RTKs and NPC1L1 are regulatory cofactors. RTKs promote viral entry by signal transduction inducing CD81–CLDN1 associationand membrane fusion [132,133] while NPC1L1 likely acts on cholesterol regulation. The factors involved in lipid and lipoprotein metabolism that relate to HCV entry step are listed in the red box including lipoprotein lipase (LpL), hepatic lipase (HL), and apolipoproteins E, B, CI, and AI (apoE, apoB, apoCI, apoAI).

Lipoprotein mediated HCV entry may be less important in cell-culture adapted HCV particles; cell culture adaptation is accompanied by a shift to higher buoyant density of the viruses *i.e.*, those not associated with TRL [125]. Indeed, the virus particles that are higher in density in cell-culture are marked by a more rapidly-infecting phenotype than the low-density viruses [126]. One of the first identified HCV entry factors was LDL receptor (LDLr), by detecting HCV RNA taken up by the hepatoma line HepG2 (Figure 2) [127]. The identification of this HCV receptor is controversial since these cells are now known to be incapable of sustaining HCV replication, and whether LDLr is involved in HCV entry or a later step in HCV infection is disputed, an issue addressed further below [120,128]. To understand how HCV utilizes molecules that play a physiological role in hepatic uptake of lipoproteins, we will review the key points of TRL metabolic pathways highlighting aspects that may share overlap with HCV entry.

TRL are assembled and produced either from enterocytes of the intestine as chylomicrons, or from hepatocytes as VLDL. These particles dock onto lipoprotein lipase (LpL) dimers tethered to the lining of the endothelium by heparan sulfate proteoglycans (HSPGs) [129]. The enzymatic activity of LpL hydrolyzes triglycerides in the core of the lipoprotein into free fatty acids that are absorbed and delivered to peripheral tissues such as skeletal muscle or adipose tissue. This enzymatic digestion of the hydrophobic core of the TRL results in diminishing the size of the lipoprotein and conversion of the lipoprotein to a cholesteryl-ester rich remnant. This lipoprotein modification is likewise accompanied by altered apolipoprotein composition, and a fraction of LpL binds to the remnants [130]. Similarly to apoE, LpL is capable of binding TRL remnant surfaces and has a cationic ligand binding domain that binds highly sulfated and negatively charged HSPGs. Hepatic lipase (HL) likewise plays a similar role as LpL, but acts preferentially on smaller VLDL.

Recent evidence has revealed that the hepatic regulation of HSPG expression and sulfation level plays a critical role in hepatocyte uptake of TRL remnants. The anatomy of the liver is marked by accessibility and exposure to the circulatory compartment. The endothelial lining of the liver has discontinuous gaps or fenestrae that act as a sieve where TRL remnants can enter the subendothelial space of Disse [134]. It is in this space that apoE on the surface of TRL remnants can encounter and interact with highly sulfated, negatively charged HSPGs present on the basolateral surface of hepatocytes. The HSPG-sequestered TRL remnants can continue to be lipolytically processed by LpL and HL even after docking. Evidence indicates that remnant internalization is a process involving multiple molecules with somewhat redundant functionality. LDLr plays a central role in remnant uptake [135], however 25%–50% of TRL remnants are still cleared in LDLr^−/−^ mice [135]. One molecule that contributes to the functional redundancy in TRL remnant uptake is in the same family of proteins as LDLr, and is termed LDL receptor related protein 1 (LRP1) [136]. In mouse studies employing genetic knockout in hepatocytes of both LDLr and LRP1, there was a marked increase in TRL remnants, whereas inactivation of LRP1 alone did not significantly impact TRL remnant clearance, indicating that physiologically LRP1 plays a more minor role in lipoprotein uptake [136]. LRP1 and HSPGs form complexes on the hepatocyte surface, acting to modulate TRL remnant binding to HSPGs [137]. Recent developments in the understanding of TRL remnant removal have focused on understanding the regulation and specificity of HSPGs in remnant uptake. Liver specific inactivation in mice of enzymes that regulate the degree of sulfation of proteoglycans on hepatocyte surfaces resulted in an accumulation of remnants over and above LDLr inactivation alone [138,139,140]. An elegant study by Stanford *et al.* demonstrated that inactivation of the hepatically expressed HSPG, syndecan 1, results in an accumulation of TRL remnants in mice [141]. Another molecule that plays a role in lipoprotein metabolism is the scavenger receptor B-I (SR-BI), which acts as a docking receptor for primarily cholesteryl-ester rich HDL to facilitate lipid transfer to the hepatocyte [142]. This is a critical role in the reverse cholesterol transport, as mammals do not have a cholesterol catabolic pathway aside from hepatic conversion of this lipid to bile acid for excretion and secretion directly into the bile duct through the ABCG5/G8 transport complex. Hepatic cholesterol levels are also regulated after billiary secretion, as cholesterol is reabsorbed through the activity of Niemann Pick C-I Like I (NPC1L1), a cholesterol transport protein principally located on the apical surface of hepatocytes, facing the bile canaliculi. This molecule also plays an active role in the intestinal absorption of cholesterol, and the specific drug ezetimibe has proven to be effective in lowering cholesterol when used in combination with a statin [143].

The aspects shared between HCV infection and lipoprotein remnant clearance abound, and evidence points to the utilization of many of the molecules that are key players in this pathway playing an important role in HCV infection, including LpL, apoE, HL, HSPGs, LDLr, SR-BI, and NPC1L1. LpL may play a dual role in the HCV life cycle. Since the enzymatic activity of this protein is to hydrolyze the triglyceride core of lipoproteins, it is likewise capable of modifying the LVP. The enzymatic digestion of HCV from serum by LpL renders HCV RNA susceptible to degradation by RNAses [144]. Andréo *et al.* determined that LpL facilitated attachment of HCV from patient serum to cells, but decreased the infectivity of HCVcc [145]. They proposed that there are two functions of LpL on HCV in this context; a structural binding function and a catalytic function. Both of these functions can impede HCV infection. Shimizu *et al.* determined that the catalytic activity of LpL reduces HCV infectivity by changing the lipid and apolipoprotein composition of the viral particle [146]. LpL mediated inhibition was confirmed by Maillard *et al.*, who found that physiological concentrations of LpL inhibits HCV infection primarily through its bridging function, and to a limited degree via its catalytic function [147]. LpL affects attachment and early steps of HCV entry, and blocks internalization of the virus particles [147]. The inhibitory effect of LpL on HCV infection may extend to the structurally similar HL, since silencing of HL in HCV producing cells results in a ~50% increase in viral titer [146]. Recent evidence supports that the host apolipoprotein CIII, which may be a LVP component, may diminish the inhibitory effect of LpL, consistent with the activity of these apolipoproteins in lipoprotein metabolism [148].

Both apoE and LpL function by binding both to TRL remnants, and to HSPGs on the hepatocyte surface. Aside from lipoprotein interactions, HCV envelope proteins are capable of binding HSPGs in the earliest attachment steps of HCV entry, as assayed using HCV pseudoparticles (HCVpp) [149,150]. Since apoE is a component of the viral particle, and is a high affinity ligand for HSPG, Jiang *et al.* examined if HSPG binding of HCV was apoE mediated [151]. Utilization of an apoE recognizing antibody pre‑ and post- attachment steps revealed that blocking apoE inhibits attachment, but not particle uptake post-attachment. Mutation of the positively charged residues in the heparan sulphate and LDLr binding domains of the apoE of HCV producing cells both diminished HCV’s capacity for infection, and rendered the apoE unable to be precipitated by the heparan sulfate analog, heparin [151]. However, similar aspects of internalization mechanisms include clustering to cholesterol-rich microdomains [152], and the requirement of tyrosine kinase activation to internalize the ligand [132,153].

The majority of evidence supports the concept that early steps of HCV entry involving viral attachment to hepatocytes is mediated by molecules that play a role in TRL remnant removal. Conceptually, this would be advantageous to the virus in utilization of an existing host pathway of particle transport from hepatocyte to hepatocyte. ApoE, present on the surface of the infectious virions, functions to mediate cell attachment through apoE binding partners [120,151]. ApoE is an amphipathic protein that is embedded on the surface of lipoproteins through hydrophobic domains and binds negatively charged cell-surface receptors through electrostatic interactions with its positively charged residues. ApoE binding partners include multiple proteins such as LDLr, although HSPGs may play an especially important role in binding apoE for TRL remnant attachment. Albecka *et al.* have recently demonstrated that while silencing expression of the LDLr gene indeed diminishes infection of Huh7 cells with HCVcc, HCV internalization occurs at a slower rate than both IDL and LDL internalization [128]. Utilizing an antibody to LDLr during HCVcc exposure markedly inhibited infection. Kinetic studies revealed strong inhibition during all steps of infection, indicating that LDLr’s uptake and metabolism, the investigators observed that treatment with this antibody indeed affected the cholesterol to cholesteryl-ester ratio in the cells. Furthermore, Huh7 cells electroporated with HCV RNA demonstrated a difference in reporter gene expression 24 h post-electroporation in the presence of LDLr antibody, suggesting that the presence of this antibody affects HCV replication [128]. Additionally, soluble LDLr that lacks the regions that tether LDLr to the cell surface and facilitate internalization of lipoproteins inhibited HCVcc but not HCVpp infection, presumably by competing with LDLr binding sites on the virion surface. These findings indicate that LDLr indeed plays an important role in HCV infection, but perhaps not a direct role at the early steps of viral attachment and entry. This is consistent with the apoE isoform not playing a major role in HCVcc infection [51,52,53,54]. More definitive experiments will need to be performed to conclusively rule out LDLr as an attachment factor for HCV infection.

SR-BI was identified as an HCV receptor due to its direct binding to HCV envelope protein E2 [154]. Since its identification, SR-BI has been confirmed to play a key role in HCV entry [155,156,157,158,159,160,161,162]. Indeed, therapeutic intervention designed to inhibit SR-BI activity, originally purposed for a cardioprotective role by increasing HDL levels [163], may prove to be a promising therapy for HCV infection [164]. Although the primary physiological role of SR-BI is in reverse cholesterol transport from HDL to hepatocytes, this protein also plays a role in TRL remnant metabolism [165,166]. Recent evidence has revealed multiple usage functions of SR-BI in HCV entry. Dao Thi *et al.* elegantly demonstrated differing functions for different HCV subpopulations distinguished by lipoprotein association using buoyant density [167]. These functions included attachment of HCV to the cells, an access function of HCV entry, and an enhancement function that boosts the infection [167]. While the attachment function of SR-BI was important for the higher-density particles and independent of HCV E2 protein, access and enhancement were important for more intermediate and lower-density HCV subpopulations, and dependent on the lipid transfer activity of SR-BI [167]. Zahid *et al.* expanded on these findings by characterizing anti-SR-BI monoclonal antibodies to find that the post-binding activity can be distinguished from the E2 binding function of SR-BI [168]. Again, the lipid transfer function of SR-BI was found to be important for this post-binding function [168]. Discovery of the critical aspect of the lipid transfer activity of SR-BI points directly to the role of lipids in HCV infection at the point of HCV entry aside from attachment.

Consistent with the role of cholesterol regulation and uptake, recent findings identified the cholesterol transporter NPC1L1 as an HCV entry factor [169]. The capability of Huh7 cells to be infected by HCVcc is diminished by genetic silencing of NPC1L1, binding by antibodies that recognize NPC1L1, and by utilizing a small chemical inhibitor of this transporter, ezetimibe. This study further implicated cholesterol metabolism in infection by utilizing an HCVcc mutant, identified by cell-culture adaptation, in the envelope protein G451R, which increases both the infectivity and the buoyant density of the virus [14,125]. Interestingly Sainz *et al.* found that this corresponded also to an increase in cholesterol content of the virion [169]. This difference in cholesterol composition is consistent with cholesterol-rich lipoproteins being higher in buoyant density than triglyceride-rich lipoproteins. Treatment of the G451 mutant is more potently neutralized with ezetimibe treatment of the target cells than wild type virus, correlating cholesterol content of the virion to usage of NPC1L1 as an entry factor [169]. Since the majority of NPC1L1 is on the apical membrane of hepatocytes, while HCV entry, apart from cell-to-cell spread, is considered to occur on the basolateral surface of the cell in proximity to the tight junctions, the role of this entry factor may occur indirectly via cholesterol regulation. 

The role of cholesterol has indeed been previously established as playing an important role in HCV entry. Depletion of cholesterol from target cell plasma membranes using the cholesterol sequestering agent methyl-beta-cyclodextrin inhibits HCV infection using both HCVcc and HCVpp [152]. The depletion of cholesterol that occurs by using methyl-beta-cyclodextrin disrupts cholesterol-rich microdomains present on the surface of hepatocyte plasma membranes. Plasma membrane proteins that localize to these lipid microdomains, such as CD81, are dependent for proper plasma membrane expression [152]. Since cholesterol-rich microdomains are critical for CD81 and this protein plays a key role at late steps of HCV infection, it is likely that the locale of HCV entry is a cholesterol-rich microdomain in the plasma membrane. Cholesterol quantity, in conjunction with sphingomyelin, has been demonstrated to dramatically affect the HCV virion fusion with liposomes, reflecting the last step of HCV entry [170]. Interestingly, eLVP generated in cell culture are capable of liposome fusion [171]. Sphingomyelinase treatment of cells, which converts sphingomyelin to ceramide, prior to infection has been demonstrated to be inhibiting [172]. Increasing the ceramide concentration in the plasma membrane diminishes CD81 surface expression, which may in turn diminish the capacity for HCV infection [172,173]. Interestingly, the density of the virus that is the most capable of HCV fusion is approximately 1.06 g/mL, the same density as LDL, the predominant cholesterol-rich lipoprotein [170,174]. While the cholesterol and sphingomyelin rich microdomains within target cells were found to be important for HCV infection, it was also shown that the enrichment of these lipids in the virion composition is also important for its infectivity [116]. Depletion of cholesterol of the virion could be resupplemented with lipid analogs dihydrocholesterol and copranastol [175]. It has been proposed that the cholesterol content of the virion may facilitate apoE interaction since this lipid facilitates precipitation with antibodies against this apolipoprotein [175]. Together these data indicate that not only proteins involved in cholesterol regulation are important for the composition and infectivity of HCV, but the lipid composition of both virion and target cell also plays a critical role.

## 6. Cholesterol and Viral Replication

**Figure 3:**
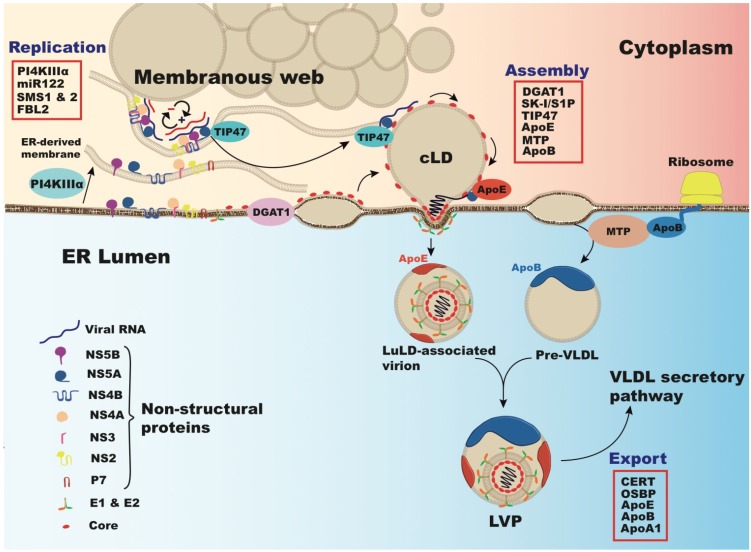
Interactions between HCV and lipid regulating factors during HCV replication and assembly. In conjunction with PI4K-IIIα (Phosphatidylinositol 4-kinase III α), viral proteins induce rearrangement of endoplasmic reticulum (ER)-derived membranes forming HCV replication complexes in modified “membranous webs” (MW) in association with cytosolic lipid droplets (cLD) [182]. Virion assembly occurs on core-enriched LDs, loaded with triglyceride by diacylglycerol acyltransferase-1 (DGAT1) [183]. LD protein Tail interacting Protein of 47 kDa (TIP47) binds RNA associated NS5A in replication complexes and mobilizes it to these LDs [184]. ApoE binds NS5A, effecting early stages of HCV assembly [119]. Packaging of the capsid occurs by viral budding into the ER lumen at sites of VLDL synthesis. VLDL synthesis is mediated by microsomal triglyceride transfer protein (MTP), which lipidates apoB and luminal ApoE-bound LDs (LuLDs) that fuse and mature to form VLDL [21]. Host factors involved in lipid metabolism and HCV replication, assembly, and production include those listed in the red box: micro-RNA 122 (miR-122), sphingomyelin synthases 1 and 2 (SMS1 & 2), F-box and leucine-rich repeat protein 2 (FBL2), subtilisin/kexin-isozyme-1 or site-1 protease (SK-I/S1P), ceramide transfer protein (CERT), oxysterol binding protein (OSBP), apolipoprotein A1 (apoA1).

Apart from HCV entry, lipids including cholesterol play critical roles in HCV replication (Figure 3). HCV replication was able to be studied extensively before other steps of the HCV life cycle by using sub-genomic HCV replicons [176]. Studies revealed that HCV replication occurs on cholesterol-rich domains within the cell as part of the HCV replication complex [177]. Inhibition of the cholesterol synthetic pathway by inhibiting the rate-limiting step 3-hydroxy-methylglutaryl CoA reductase completely disrupted HCV replication [80]. Aside from cholesterol synthesis, this could also be due to an earlier branch point of this synthetic pathway that functions to add prenyl groups to proteins, namely geranylgeranylation. It was demonstrated that the geranylgeranylation of host protein FBL2 binds to HCV nonstructural protein 5A (NS5A), playing a critical role in HCV replication [178]. The presence of HCV replication complexes in turn functions to elevate the levels of enzymes involved in cholesterol and fatty acid synthesis by activation of SREBPs [33,179]. Another transcriptional regulator of cholesterol metabolism that is activated by the bile acid catabolic products of cholesterol is the farnesoid X receptor (FXR). Bile acids were interestingly found to increase HCV replication through FXR activity [180], though this may be genotype specific [181].

Recent evidence suggests that not only cholesterol synthesis, but also sphingolipid synthesis contributes to HCV replication. HCV nonstructural proteins function to modify the endoplasmic reticulum and cytosolic lipid droplets to form double membrane vesicles containing HCV replication complexes where viral RNA is protected from host RNAses and sensors of the innate immunity that would respond to double-stranded RNA [182,185]. The replication complexes utilize cytosolic lipid droplets (LDs) as a platform where genome replication occurs in conjunction with viral assembly [186]. The lipid composition of replication complexes is not completely established, but it is known that HCV replication occurs on detergent resistant membranes, indicating that they are likely rich in both cholesterol and sphingolipids [177,187]. HCV infection induces the synthesis of sphingomyelin by increasing the expression of the genes encoding sphingomyelin synthases 1 and 2 [188]. Indeed, inhibition of sphingolipid synthesis dramatically modulates HCV replication [188,189]. Interestingly, genotype specificity appears to play a role in the RNA dependent RNA polymerase NS5B being bound and activated by sphingomyelin through enhancement of its binding of the template RNA [190]. While it is not completely understood how the lipid composition of these specialized compartments are formed, recent evidence indicates a key role for phosphotidylinositol 4-kinase, subtype III alpha (PI4KIIIα) [191]. This protein was identified through a siRNA screen of kinases that would affect HCV replication, and interestingly, HCV NS5A is able to bind and enhance the kinase activity of this protein.

An important element of both HCV replication and cholesterol metabolism is the liver-specific miR-122, which is the predominant miRNA expressed in the liver and plays a key role in hepatic fatty acid and cholesterol metabolism [89]. Deletion of miR-122 results in hepatosteatosis and diminished circulating cholesterol and lipoprotein levels, a phenotype also reflected in HCV infected patients [192]. Also as in HCV infection, this phenotype may in part be mediated through transcriptional modulation of microsomal triglyceride transferase, an enzyme required for lipoprotein production [43,44,193]. Furthermore, the absence of miR-122 leads to the development of hepatocellular carcinoma in mice [193]. This miRNA is also unique in that it directly binds to the HCV genome and acts to boost its replication [194]. In fact, this is one of a few factors that confer permissibility of non-hepatic cells for robust HCV infection [52]. 

The mechanism of miR-122 enhancement of HCV infection is at least in part due to the stabilization of the genome by forming a protein complex with host Argonaute 2, protecting the genome from exonucleases [195]. The therapeutic possibility of miR-122 as a target has been explored in chimpanzees with HCV infection, yielding promising results [90]. It is an intriguing hypothesis that the pathology of HCV relating to lipids could be due to the sequestration of miR-122 into these complexes, but the physiological levels of HCV RNA during infection are not likely sufficient to exert such an effect and there is no *in vivo* correlation with HCV RNA and miR-122 levels [196]. The interplay between this important regulator of cholesterol and lipid metabolism and the pathologic effects of HCV infection remain an interesting aspect of HCV research.

## 7. Lipoproteins, Lipids and Cholesterol in Virus Production and Secretion

LDs are the organelles specialized in the storage and release of lipids. LDs are surrounded by proteins belonging to the PAT family like the adipose related protein (ADRP), the tail interacting protein of 47kDa (TIP47), and proteins involved in lipid biogenesis [197]. The protein and lipid content of the LD fluctuates in response to the metabolic needs of the cell induced by cellular lipases [198]. HCV infection also influences the proteins associating with LDs. Following HCV core over-expression LDs are enriched in TIP47 and depleted of ADRP [199]. Recent evidence indicates that TIP47 interacts with NS5A, which leads to productive HCV secretion [184]. Core-associated LDs form the viral assembly platforms were the nascent virions are produced [185]. Indeed, viral assembly is initiated by the association of cleaved core protein to cytosolic LD displacing ADRP. There is a balance between the amount of core on the LD surface and virus production [200,201,202,203]. This active process is mediated by the interaction between the diacylglycerol acyltransferase-1 (DGAT-1) and HCV core. DGAT1, an enzyme involved in triglyceride synthesis and luminal LD maturation, targets core to LDs. Its absence or inhibition leads to the inhibition of viral assembly and production [183]. Modifications affecting LD physiology, including composition, size, and apoB localization also display dramatic effects on HCV assembly and secretion [204]. Besides their major role in HCV replication [205], cyclophilins also impact HCV secretion. Inhibition of cyclophilins by inhibitors or gene silencing modifies cellular protein trafficking, LD size, and apoB subcellular localization [206]. Furthermore, the SREBP protease subtilisin/kexin-isozyme-1/site 1protease (SKI1/S1P) affects LD formation altering lipid and ADRP content as well as size. Consequently, HCV infection is severely impaired by the use of a specific inhibitor or gene silencing of SK-I/S1P [207]. 

Comparison of the buoyant density of intracellular and extracellular HCVcc particles suggests that the virus associates with VLDL-like structures during its egress, suggesting an intimate link between the VLDL synthesis, maturation and secretion process and HCV infectious virion production. Indeed, actors of the VLDL synthesis mechanism, like apoB, MTP, apoE and apoA1 have been shown to be direct players in the virion production mechanism. In brief, blocking the VLDL synthesis by RNA interference towards apoB or apoE or by chemical inhibitors of the MTP or the acyl-coA-synthase 3, blocks HCV production without affecting viral replication [60,92,108,109,119,208]. It has been reported that nonstructural viral proteins of the subgenomic replicon decreases apoB secretion via MTP inhibition, which has a consequence on VLDL synthesis [43], a phenomenon which may be redundant also with HCV core protein in the core-transgenic mouse model [209]. These observations correlate well with MTP and apoB decrease in HCV genotype 3 infected patients [44,210]. Moreover, apoB and apoE inhibition leads to a decrease in intracellular HCV infectivity suggesting a role of both apolipoproteins in HCV maturation [109].

In parallel to its structural role in VLDL formation, apoB has been shown to be required for HCV glycoprotein secretion, albeit in a non-hepatic and non-infectious context [117]. However, a direct active role of apoB in HCV production has not been clearly proven in the classical Huh-7 cell culture model [211]. The exact role of apoB thus remains elusive. Consistent with the critical nature of lipoproteins in the HCV life cycle, Steenbergen *et al.* observed that the humanized mouse model is only capable of sustaining HCV infection if the human hepatocytes repopulated the mouse to the extent that the lipoprotein profile reflected the lipoprotein distribution observed in humans [212].

Among the apolipoproteins involved in HCV assembly and production, apoE plays a major role. ApoE depletion displays a more dramatic effect than apoB or apoA1 in HCVcc production. This is likely due to an important role of apoE in HCV assembly [53,108,119,211]. ApoE interacts with NS5A during HCV infection [51,119]. Since NS5A is a component of the HCV replication complex, and is assumed to bring the replication complex to the LD [185], it is likely that the NS5A-apoE interaction helps the building of the viral assembly platforms. Moreover, Coller *et al.* observed that tetracystein-tagged core co-transports with apoE-GFP [213].

Viruses of the *Flaviviridae* family use the secretory pathway of the host cell for their egress. In the case of HCV, the secretion pathway is tightly associated with host factors of lipoprotein metabolism, suggesting that the virus diverts the VLDL synthesis mechanism for its selective advantage. It has been shown that apoE plays a crucial role in viral maturation and secretion. Furthermore, the interaction of the oxysterol binding protein (OSBP) with NS5A could play a direct role in HCV secretion. Indeed, it has been shown that inhibiting phosphorylation of OSBP and the ceramide transfer protein (CERT) by protein kinase D (PKD), a protein recruited at the trans-Golgi network, has dramatic effects on HCV maturation and secretion likely due to a subsequent decrease in sphingolipid biosynthesis [214,215]. However, in the early phases of HCV infection, an increase in sphingolipid synthesis is observed, as a physiologic response to viral replication and membrane reorganization [216]. An extensive siRNA screen has been recently performed and demonstrated that most of the host cell proteins involved in the HCV secretion are part of the classical trafficking pathway, including the Golgi recycling endosomes, microtubules, VAMP1 secretory vesicles, and apoE which co-trafficks with core protein [213].

HCV infection is unique in its dependency and modification of host lipid and lipoprotein metabolism. The interactions with these pathways contribute to the pathogenesis of the disease, and are likely to play important roles in vaccine development, and therapeutic considerations. Furthermore, the inclusion of lipid modulating therapies along with DAAs may increase their effectiveness. Since an increasing number of patients are administered lipid modulating drugs, it is essential to further understand virus/host interactions that affect lipid and lipoprotein metabolism to inform and improve treatment options.

## References

[1] Aghemo A., De Francesco R. (2013). New horizons in Hepatitis C antiviral therapy with direct-acting antivirals. Hepatology.

[2] Kanwal F., Hoang T., Kramer J.R., Asch S.M., Goetz M.B., Zeringue A., Richardson P., El-Serag H.B. (2011). Increasing prevalence of HCC and cirrhosis in patients with chronic hepatitis C virus infection. Gastroenterology.

[3] Hezode C., Forestier N., Dusheiko G., Ferenci P., Pol S., Goeser T., Bronowicki J.P., Bourliere M., Gharakhanian S., Bengtsson L. (2009). Telaprevir and peginterferon with or without ribavirin for chronic HCV infection. N. Engl. J. Med..

[4] McHutchison J.G., Everson G.T., Gordon S.C., Jacobson I.M., Sulkowski M., Kauffman R., McNair L., Alam J., Muir A.J. (2009). Telaprevir with peginterferon and ribavirin for chronic HCV genotype 1 infection. N. Engl. J. Med..

[5] Poordad F., McCone J., Bacon B.R., Bruno S., Manns M.P., Sulkowski M.S., Jacobson I.M., Reddy K.R., Goodman Z.D., Boparai N. (2011). Boceprevir for untreated chronic HCV genotype 1 infection. N. Engl. J. Med..

[6] Bacon B.R., Gordon S.C., Lawitz E., Marcellin P., Vierling J.M., Zeuzem S., Poordad F., Goodman Z.D., Sings H.L., Boparai N. (2011). Boceprevir for previously treated chronic HCV genotype 1 infection. N. Engl. J. Med..

[7] McHutchison J.G., Manns M.P., Muir A.J., Terrault N.A., Jacobson I.M., Afdhal N.H., Heathcote E.J., Zeuzem S., Reesink H.W., Garg J. (2010). Telaprevir for previously treated chronic HCV infection. N. Engl. J. Med..

[8] Dienes H.P., Popper H., Arnold W., Lobeck H. (1982). Histologic observations in human hepatitis non-A, non-B. Hepatology.

[9] Thomssen R., Bonk S., Propfe C., Heermann K.H., Kochel H.G., Uy A. (1992). Association of hepatitis C virus in human sera with beta-lipoprotein. Med. Microbiol. Immunol..

[10] Thomssen R., Bonk S., Thiele A. (1993). Density heterogeneities of hepatitis C virus in human sera due to the binding of beta-lipoproteins and immunoglobulins. Med. Microbiol. Immunol..

[11] Diaz O., Delers F., Maynard M., Demignot S., Zoulim F., Chambaz J., Trepo C., Lotteau V., Andre P. (2006). Preferential association of Hepatitis C virus with apolipoprotein B48-containing lipoproteins. J. Gen. Virol..

[12] Felmlee D.J., Sheridan D.A., Bridge S.H., Nielsen S.U., Milne R.W., Packard C.J., Caslake M.J., McLauchlan J., Toms G.L., Neely R.D. (2010). Intravascular transfer contributes to postprandial increase in numbers of very-low-density hepatitis C virus particles. Gastroenterology.

[13] Scholtes C., Ramiere C., Rainteau D., Perrin-Cocon L., Wolf C., Humbert L., Carreras M., Guironnet-Paquet A., Zoulim F., Bartenschlager R. (2012). High plasma level of nucleocapsid-free envelope glycoprotein-positive lipoproteins in hepatitis C patients. Hepatology.

[14] Grove J., Nielsen S., Zhong J., Bassendine M.F., Drummer H.E., Balfe P., McKeating J.A. (2008). Identification of a residue in hepatitis C virus E2 glycoprotein that determines scavenger receptor BI and CD81 receptor dependency and sensitivity to neutralizing antibodies. J. Virol..

[15] Prentoe J., Jensen T.B., Meuleman P., Serre S.B., Scheel T.K., Leroux-Roels G., Gottwein J.M., Bukh J. (2011). Hypervariable region 1 differentially impacts viability of hepatitis C virus strains of genotypes 1 to 6 and impairs virus neutralization. J. Virol..

[16] Fafi-Kremer S., Fofana I., Soulier E., Carolla P., Meuleman P., Leroux-Roels G., Patel A.H., Cosset F.L., Pessaux P., Doffoel M. (2010). Viral entry and escape from antibody-mediated neutralization influence hepatitis C virus reinfection in liver transplantation. J. Exp. Med..

[17] Fofana I., Fafi-Kremer S., Carolla P., Fauvelle C., Zahid M.N., Turek M., Heydmann L., Cury K., Hayer J., Combet C. (2012). Mutations that alter use of hepatitis C virus cell entry factors mediate escape from neutralizing antibodies. Gastroenterology.

[18] Bickel P.E., Tansey J.T., Welte M.A. (2009). PAT proteins, an ancient family of lipid droplet proteins that regulate cellular lipid stores. Biochim. Biophys. Acta.

[19] Brasaemle D.L. (2007). Thematic review series: Adipocyte biology. The perilipin family of structural lipid droplet proteins: Stabilization of lipid droplets and control of lipolysis. J. Lipid. Res..

[20] Dallinga-Thie G.M., Franssen R., Mooij H.L., Visser M.E., Hassing H.C., Peelman F., Kastelein J.J., Peterfy M., Nieuwdorp M. (2010). The metabolism of triglyceride-rich lipoproteins revisited: New players, new insight. Atherosclerosis.

[21] Williams K.J. (2008). Molecular processes that handle—and mishandle—dietary lipids. J. Clin. Invest..

[22] Andre P., Komurian-Pradel F., Deforges S., Perret M., Berland J.L., Sodoyer M., Pol S., Brechot C., Paranhos-Baccala G., Lotteau V. (2002). Characterization of low- and very-low-density hepatitis C virus RNA-containing particles. J. Virol..

[23] Nielsen S.U., Bassendine M.F., Burt A.D., Martin C., Pumeechockchai W., Toms G.L. (2006). Association between hepatitis C virus and very-low-density lipoprotein (VLDL)/LDL analyzed in iodixanol density gradients. J. Virol..

[24] Rubbia-Brandt L., Quadri R., Abid K., Giostra E., Male P.J., Mentha G., Spahr L., Zarski J.P., Borisch B., Hadengue A. (2000). Hepatocyte steatosis is a cytopathic effect of hepatitis C virus genotype 3. J. Hepatol..

[25] Serfaty L., Andreani T., Giral P., Carbonell N., Chazouilleres O., Poupon R. (2001). Hepatitis C virus induced hypobetalipoproteinemia: A possible mechanism for steatosis in chronic hepatitis C. J. Hepatol..

[26] Fujino T., Nakamuta M., Yada R., Aoyagi Y., Yasutake K., Kohjima M., Fukuizumi K., Yoshimoto T., Harada N., Yada M. (2010). Expression profile of lipid metabolism-associated genes in hepatitis C virus-infected human liver. Hepatol. Res..

[27] Nakamuta M., Yada R., Fujino T., Yada M., Higuchi N., Tanaka M., Miyazaki M., Kohjima M., Kato M., Yoshimoto T. (2009). Changes in the expression of cholesterol metabolism-associated genes in HCV-infected liver: A novel target for therapy?. Int. J. Mol. Med..

[28] Su A.I., Pezacki J.P., Wodicka L., Brideau A.D., Supekova L., Thimme R., Wieland S., Bukh J., Purcell R.H., Schultz P.G. (2002). Genomic analysis of the host response to hepatitis C virus infection. Proc. Natl. Acad. Sci. USA.

[29] Chang M.L., Yeh C.T., Chen J.C., Huang C.C., Lin S.M., Sheen I.S., Tai D.I., Chu C.M., Lin W.P., Chang M.Y. (2008). Altered expression patterns of lipid metabolism genes in an animal model of HCV core-related, nonobese, modest hepatic steatosis. BMC Genomics.

[30] Lerat H., Kammoun H.L., Hainault I., Merour E., Higgs M.R., Callens C., Lemon S.M., Foufelle F., Pawlotsky J.M. (2009). Hepatitis C virus proteins induce lipogenesis and defective triglyceride secretion in transgenic mice. J. Biol. Chem..

[31] Oem J.K., Jackel-Cram C., Li Y.P., Zhou Y., Zhong J., Shimano H., Babiuk L.A., Liu Q. (2008). Activation of sterol regulatory element-binding protein 1c and fatty acid synthase transcription by hepatitis C virus non-structural protein 2. J. Gen. Virol..

[32] Park C.Y., Jun H.J., Wakita T., Cheong J.H., Hwang S.B. (2009). Hepatitis C virus nonstructural 4B protein modulates sterol regulatory element-binding protein signaling via the AKT pathway. J. Biol. Chem..

[33] Waris G., Felmlee D.J., Negro F., Siddiqui A. (2007). Hepatitis C virus induces proteolytic cleavage of sterol regulatory element binding proteins and stimulates their phosphorylation via oxidative stress. J. Virol..

[34] McPherson S., Jonsson J.R., Barrie H.D., O'Rourke P., Clouston A.D., Powell E.E. (2008). Investigation of the role of SREBP-1c in the pathogenesis of HCV-related steatosis. J. Hepatol..

[35] Lambert J.E., Bain V.G., Ryan E.A., Thomson A.B., Clandinin M.T. (2012). Elevated lipogenesis and diminished cholesterol synthesis in patients with hepatitis C viral infection compared to healthy humans. Hepatology.

[36] Sharma P., Balan V., Hernandez J., Rosati M., Williams J., Rodriguez-Luna H., Schwartz J., Harrison E., Anderson M., Byrne T. (2004). Hepatic steatosis in hepatitis C virus genotype 3 infection: Does it correlate with body mass index, fibrosis, and HCV risk factors?. Dig. Dis. Sci..

[37] Thomopoulos K.C., Arvaniti V., Tsamantas A.C., Dimitropoulou D., Gogos C.A., Siagris D., Theocharis G.J., Labropoulou-Karatza C. (2006). Prevalence of liver steatosis in patients with chronic hepatitis B: A study of associated factors and of relationship with fibrosis. Eur. J. Gastroenterol. Hepatol..

[38] Adinolfi L.E., Gambardella M., Andreana A., Tripodi M.F., Utili R., Ruggiero G. (2001). Steatosis accelerates the progression of liver damage of chronic hepatitis C patients and correlates with specific HCV genotype and visceral obesity. Hepatology.

[39] Adinolfi L.E., Utili R., Andreana A., Tripodi M.F., Marracino M., Gambardella M., Giordano M., Ruggiero G. (2001). Serum HCV RNA levels correlate with histological liver damage and concur with steatosis in progression of chronic hepatitis C. Dig. Dis. Sci..

[40] Hofer H., Bankl H.C., Wrba F., Steindl-Munda P., Peck-Radosavljevic M., Osterreicher C., Mueller C., Gangl A., Ferenci P. (2002). Hepatocellular fat accumulation and low serum cholesterol in patients infected with HCV-3a. Am. J. Gastroenterol..

[41] Marzouk D., Sass J., Bakr I., El Hosseiny M., Abdel-Hamid M., Rekacewicz C., Chaturvedi N., Mohamed M.K., Fontanet A. (2007). Metabolic and cardiovascular risk profiles and hepatitis C virus infection in rural Egypt. Gut.

[42] Clark P.J., Thompson A.J., Vock D.M., Kratz L.E., Tolun A.A., Muir A.J., McHutchison J.G., Subramanian M., Millington D.M., Kelley R.I. (2012). Hepatitis C virus selectively perturbs the distal cholesterol synthesis pathway in a genotype-specific manner. Hepatology.

[43] Domitrovich A.M., Felmlee D.J., Siddiqui A. (2005). Hepatitis C virus nonstructural proteins inhibit apolipoprotein B100 secretion. J. Biol. Chem..

[44] Mirandola S., Realdon S., Iqbal J., Gerotto M., Dal Pero F., Bortoletto G., Marcolongo M., Vario A., Datz C., Hussain M.M. (2006). Liver microsomal triglyceride transfer protein is involved in hepatitis C liver steatosis. Gastroenterology.

[45] Mancone C., Montaldo C., Santangelo L., Di Giacomo C., Costa V., Amicone L., Ippolito G., Pucillo L.P., Alonzi T., Tripodi M. (2012). Ferritin heavy chain is the host factor responsible for HCV-induced inhibition of apoB-100 production and is required for efficient viral infection. J. Proteome Res..

[46] Enjoji M., Kohjima M., Kotoh K., Nakamuta M. (2012). Metabolic disorders and steatosis in patients with chronic hepatitis C: Metabolic strategies for antiviral treatments. Int. J. Hepatol..

[47] Dai C.Y., Chuang W.L., Ho C.K., Hsieh M.Y., Huang J.F., Lee L.P., Hou N.J., Lin Z.Y., Chen S.C., Hsieh M.Y. (2008). Associations between hepatitis C viremia and low serum triglyceride and cholesterol levels: A community-based study. J. Hepatol..

[48] Nishimura M., Yamamoto H., Yoshida T., Seimiya M., Sawabe Y., Matsushita K., Umemura H., Sogawa K., Takizawa H., Yokosuka O. (2011). Decreases in the serum VLDL-TG/non-VLDL-TG ratio from early stages of chronic hepatitis C: Alterations in TG-rich lipoprotein levels. PLoS One.

[49] Merz A., Long G., Hiet M.S., Brugger B., Chlanda P., Andre P., Wieland F., Krijnse-Locker J., Bartenschlager R. (2011). Biochemical and morphological properties of hepatitis C virus particles and determination of their lipidome. J. Biol. Chem..

[50] Akazawa D., Morikawa K., Omi N., Takahashi H., Nakamura N., Mochizuki H., Date T., Ishii K., Suzuki T., Wakita T. (2011). Production and characterization of HCV particles from serum-free culture. Vaccine.

[51] Cun W., Jiang J., Luo G. (2010). The C-terminal alpha-helix domain of apolipoprotein E is required for interaction with nonstructural protein 5A and assembly of hepatitis C virus. J. Virol..

[52] Da Costa D., Turek M., Felmlee D.J., Girardi E., Pfeffer S., Long G., Bartenschlager R., Zeisel M.B., Baumert T.F. (2012). Reconstitution of the entire hepatitis C virus life cycle in nonhepatic cells. J. Virol..

[53] Hishiki T., Shimizu Y., Tobita R., Sugiyama K., Ogawa K., Funami K., Ohsaki Y., Fujimoto T., Takaku H., Wakita T. (2010). Infectivity of hepatitis C virus is influenced by association with apolipoprotein E isoforms. J. Virol..

[54] Long G., Hiet M.S., Windisch M.P., Lee J.Y., Lohmann V., Bartenschlager R. (2011). Mouse hepatic cells support assembly of infectious hepatitis C virus particles. Gastroenterology.

[55] Price D.A., Bassendine M.F., Norris S.M., Golding C., Toms G.L., Schmid M.L., Morris C.M., Burt A.D., Donaldson P.T. (2006). Apolipoprotein epsilon3 allele is associated with persistent hepatitis C virus infection. Gut.

[56] Kuhlmann I., Minihane A.M., Huebbe P., Nebel A., Rimbach G. (2010). Apolipoprotein E genotype and hepatitis C, HIV and herpes simplex disease risk: A literature review. Lipids Health Dis..

[57] Wozniak M.A., Itzhaki R.F., Faragher E.B., James M.W., Ryder S.D., Irving W.L. (2002). Apolipoprotein E-epsilon 4 protects against severe liver disease caused by hepatitis C virus. Hepatology.

[58] Li H., Liu Z., Han Q., Li Y., Chen J. (2006). Association of genetic polymorphism of low-density lipoprotein receptor with chronic viral hepatitis C infection in Han Chinese. J. Med. Virol..

[59] Napolitano M., Giuliani A., Alonzi T., Mancone C., D'Offizi G., Tripodi M., Bravo E. (2007). Very low density lipoprotein and low density lipoprotein isolated from patients with hepatitis C infection induce altered cellular lipid metabolism. J. Med. Virol..

[60] Mancone C., Steindler C., Santangelo L., Simonte G., Vlassi C., Longo M.A., D'Offizi G., Di Giacomo C., Pucillo L.P., Amicone L. (2011). Hepatitis C virus production requires apolipoprotein A-I and affects its association with nascent low-density lipoproteins. Gut.

[61] Kim E., Li K., Lieu C., Tong S., Kawai S., Fukutomi T., Zhou Y., Wands J., Li J. (2008). Expression of apolipoprotein C-IV is regulated by Ku antigen/peroxisome proliferator-activated receptor gamma complex and correlates with liver steatosis. J. Hepatol..

[62] Rowell J., Thompson A.J., Guyton J.R., Lao X.Q., McHutchison J.G., McCarthy J.J., Patel K. (2012). Serum apolipoprotein C-III is independently associated with chronic hepatitis C infection and advanced fibrosis. Hepatol. Int..

[63] Poynard T., Ratziu V., McHutchison J., Manns M., Goodman Z., Zeuzem S., Younossi Z., Albrecht J. (2003). Effect of treatment with peginterferon or interferon alfa-2b and ribavirin on steatosis in patients infected with hepatitis C. Hepatology.

[64] Westin J., Lagging M., Dhillon A.P., Norkrans G., Romero A.I., Pawlotsky J.M., Zeuzem S., Schalm S.W., Verheij-Hart E., Negro F. (2007). Impact of hepatic steatosis on viral kinetics and treatment outcome during antiviral treatment of chronic HCV infection. J. Viral. Hepat..

[65] Zeuzem S., Hultcrantz R., Bourliere M., Goeser T., Marcellin P., Sanchez-Tapias J., Sarrazin C., Harvey J., Brass C., Albrecht J. (2004). Peginterferon alfa-2b plus ribavirin for treatment of chronic hepatitis C in previously untreated patients infected with HCV genotypes 2 or 3. J. Hepatol..

[66] Corey K.E., Kane E., Munroe C., Barlow L.L., Zheng H., Chung R.T. (2009). Hepatitis C virus infection and its clearance alter circulating lipids: Implications for long-term follow-up. Hepatology.

[67] Gopal K., Johnson T.C., Gopal S., Walfish A., Bang C.T., Suwandhi P., Pena-Sahdala H.N., Clain D.J., Bodenheimer H.C., Min A.D. (2006). Correlation between beta-lipoprotein levels and outcome of hepatitis C treatment. Hepatology.

[68] Ramcharran D., Wahed A.S., Conjeevaram H.S., Evans R.W., Wang T., Belle S.H., Yee L.J. (2010). Associations between serum lipids and hepatitis C antiviral treatment efficacy. Hepatology.

[69] Adinolfi L.E., Restivo L., Zampino R., Guerrera B., Lonardo A., Ruggiero L., Riello F., Loria P., Florio A. (2012). Chronic HCV infection is a risk of atherosclerosis. Role of HCV and HCV-related steatosis. Atherosclerosis.

[70] Akuta N., Suzuki F., Kawamura Y., Yatsuji H., Sezaki H., Suzuki Y., Hosaka T., Kobayashi M., Kobayashi M., Arase Y. (2007). Predictive factors of early and sustained responses to peginterferon plus ribavirin combination therapy in Japanese patients infected with hepatitis C virus genotype 1b: Amino acid substitutions in the core region and low-density lipoprotein cholesterol levels. J. Hepatol..

[71] Sheridan D.A., Bridge S.H., Felmlee D.J., Crossey M.M., Thomas H.C., Taylor-Robinson S.D., Toms G.L., Neely R.D., Bassendine M.F. (2012). Apolipoprotein-E and hepatitis C lipoviral particles in genotype 1 infection: Evidence for an association with interferon sensitivity. J. Hepatol..

[72] Bridge S.H., Sheridan D.A., Felmlee D.J., Nielsen S.U., Thomas H.C., Taylor-Robinson S.D., Neely R.D., Toms G.L., Bassendine M.F. (2011). Insulin resistance and low-density apolipoprotein B-associated lipoviral particles in hepatitis C virus genotype 1 infection. Gut.

[73] Li J.H., Lao X.Q., Tillmann H.L., Rowell J., Patel K., Thompson A., Suchindran S., Muir A.J., Guyton J.R., Gardner S.D. (2010). Interferon-lambda genotype and low serum low-density lipoprotein cholesterol levels in patients with chronic hepatitis C infection. Hepatology.

[74] Romero-Gomez M., Diago M., Andrade R.J., Calleja J.L., Salmeron J., Fernandez-Rodriguez C.M., Sola R., Garcia-Samaniego J., Herrerias J.M., De la Mata M. (2009). Treatment of insulin resistance with metformin in naive genotype 1 chronic hepatitis C patients receiving peginterferon alfa-2a plus ribavirin. Hepatology.

[75] Kohjima M., Enjoji M., Yoshimoto T., Yada R., Fujino T., Aoyagi Y., Fukushima N., Fukuizumi K., Harada N., Yada M. (2013). Add-on therapy of pitavastatin and eicosapentaenoic acid improves outcome of peginterferon plus ribavirin treatment for chronic hepatitis C. J. Med. Virol..

[76] Chojkier M., Elkhayat H., Sabry D., Donohue M., Buck M. (2012). Pioglitazone decreases hepatitis C viral load in overweight, Treatment naive, Genotype 4 infected-patients: A pilot study. PLoS One.

[77] Harrison S.A., Rossaro L., Hu K.Q., Patel K., Tillmann H., Dhaliwal S., Torres D.M., Koury K., Goteti V.S., Noviello S. (2010). Serum cholesterol and statin use predict virological response to peginterferon and ribavirin therapy. Hepatology.

[78] Atsukawa M., Tsubota A., Kondo C., Itokawa N., Narahara Y., Nakatsuka K., Hashimoto S., Fukuda T., Matsushita Y., Kidokoro H. (2013). Combination of fluvastatin with pegylated interferon/ribavirin therapy reduces viral relapse in chronic hepatitis C infected with HCV genotype 1b. J. Gastroenterol. Hepatol..

[79] Rao G.A., Pandya P.K. (2011). Statin therapy improves sustained virologic response among diabetic patients with chronic hepatitis C. Gastroenterology.

[80] Ye J., Wang C., Sumpter R., Brown M.S., Goldstein J.L., Gale M. (2003). Disruption of hepatitis C virus RNA replication through inhibition of host protein geranylgeranylation. Proc. Natl. Acad. Sci. USA.

[81] Bader T., Fazili J., Madhoun M., Aston C., Hughes D., Rizvi S., Seres K., Hasan M. (2008). Fluvastatin inhibits hepatitis C replication in humans. Am. J. Gastroenterol..

[82] Forde K.A., Law C., O'Flynn R., Kaplan D.E. (2009). Do statins reduce hepatitis C RNA titers during routine clinical use?. World J. Gastroenterol..

[83] Milazzo L., Meroni L., Galazzi M., Cesari M., Caramma I., Marchetti G., Galli M., Antinori S. (2009). Does fluvastatin favour HCV replication *in vivo*? A pilot study on HIV-HCV coinfected patients. J. Viral. Hepatitis.

[84] O'Leary J.G., Chan J.L., McMahon C.M., Chung R.T. (2007). Atorvastatin does not exhibit antiviral activity against HCV at conventional doses: A pilot clinical trial. Hepatology.

[85] McHutchison J.G., Lawitz E.J., Shiffman M.L., Muir A.J., Galler G.W., McCone J., Nyberg L.M., Lee W.M., Ghalib R.H., Schiff E.R. (2009). Peginterferon alfa-2b or alfa-2a with ribavirin for treatment of hepatitis C infection. N. Engl. J. Med..

[86] Milazzo L., Caramma I., Mazzali C., Cesari M., Olivetti M., Galli M., Antinori S. (2010). Fluvastatin as an adjuvant to pegylated interferon and ribavirin in HIV/hepatitis C virus genotype 1 co-infected patients: An open-label randomized controlled study. J. Antimicrob. Chemother..

[87] Sezaki H., Suzuki F., Akuta N., Yatsuji H., Hosaka T., Kobayashi M., Suzuki Y., Arase Y., Ikeda K., Miyakawa Y. (2009). An open pilot study exploring the efficacy of fluvastatin, pegylated interferon and ribavirin in patients with hepatitis C virus genotype 1b in high viral loads. Intervirology.

[88] Raal F.J., Santos R.D., Blom D.J., Marais A.D., Charng M.J., Cromwell W.C., Lachmann R.H., Gaudet D., Tan J.L., Chasan-Taber S. (2010). Mipomersen, an apolipoprotein B synthesis inhibitor, for lowering of LDL cholesterol concentrations in patients with homozygous familial hypercholesterolaemia: A randomised, double-blind, placebo-controlled trial. Lancet.

[89] Esau C., Davis S., Murray S.F., Yu X.X., Pandey S.K., Pear M., Watts L., Booten S.L., Graham M., McKay R. (2006). miR-122 regulation of lipid metabolism revealed by *in vivo* antisense targeting. Cell Metab..

[90] Lanford R.E., Hildebrandt-Eriksen E.S., Petri A., Persson R., Lindow M., Munk M.E., Kauppinen S., Orum H. (2010). Therapeutic silencing of microRNA-122 in primates with chronic hepatitis C virus infection. Science.

[91] Goldwasser J., Cohen P.Y., Lin W., Kitsberg D., Balaguer P., Polyak S.J., Chung R.T., Yarmush M.L., Nahmias Y. (2011). Naringenin inhibits the assembly and long-term production of infectious hepatitis C virus particles through a PPAR-mediated mechanism. J. Hepatol..

[92] Nahmias Y., Goldwasser J., Casali M., van Poll D., Wakita T., Chung R.T., Yarmush M.L. (2008). Apolipoprotein B-dependent hepatitis C virus secretion is inhibited by the grapefruit flavonoid naringenin. Hepatology.

[93] Ciesek S., von Hahn T., Colpitts C.C., Schang L.M., Friesland M., Steinmann J., Manns M.P., Ott M., Wedemeyer H., Meuleman P. (2011). The green tea polyphenol, epigallocatechin-3-gallate, Inhibits hepatitis C virus entry. Hepatology.

[94] Calland N., Albecka A., Belouzard S., Wychowski C., Duverlie G., Descamps V., Hober D., Dubuisson J., Rouille Y., Seron K. (2012). (−)-Epigallocatechin-3-gallate is a new inhibitor of hepatitis C virus entry. Hepatology.

[95] Lin Y.T., Wu Y.H., Tseng C.K., Lin C.K., Chen W.C., Hsu Y.C., Lee J.C. (2013). Green tea phenolic epicatechins inhibit hepatitis C virus replication via cycloxygenase-2 and attenuate virus-induced inflammation. PLoS One.

[96] Kaito M., Watanabe S., Tsukiyama-Kohara K., Yamaguchi K., Kobayashi Y., Konishi M., Yokoi M., Ishida S., Suzuki S., Kohara M. (1994). Hepatitis C virus particle detected by immunoelectron microscopic study. J. Gen. Virol..

[97] Bradley D., McCaustland K., Krawczynski K., Spelbring J., Humphrey C., Cook E.H. (1991). Hepatitis C virus: Buoyant density of the factor VIII-derived isolate in sucrose. J. Med. Virol..

[98] Bradley D.W., McCaustland K.A., Cook E.H., Schable C.A., Ebert J.W., Maynard J.E. (1985). Posttransfusion non-A, non-B hepatitis in chimpanzees. Physicochemical evidence that the tubule-forming agent is a small, enveloped virus. Gastroenterology.

[99] He L.F., Alling D., Popkin T., Shapiro M., Alter H.J., Purcell R.H. (1987). Determining the size of non-A, non-B hepatitis virus by filtration. J. Infect. Dis..

[100] Kanto T., Hayashi N., Takehara T., Hagiwara H., Mita E., Naito M., Kasahara A., Fusamoto H., Kamada T. (1995). Density analysis of hepatitis C virus particle population in the circulation of infected hosts: Implications for virus neutralization or persistence. J. Hepatol..

[101] Pumeechockchai W., Bevitt D., Agarwal K., Petropoulou T., Langer B.C., Belohradsky B., Bassendine M.F., Toms G.L. (2002). Hepatitis C virus particles of different density in the blood of chronically infected immunocompetent and immunodeficient patients: Implications for virus clearance by antibody. J. Med. Virol..

[102] Nielsen S.U., Bassendine M.F., Martin C., Lowther D., Purcell P.J., King B.J., Neely D., Toms G.L. (2008). Characterization of hepatitis C RNA-containing particles from human liver by density and size. J. Gen. Virol..

[103] Hijikata M., Shimizu Y.K., Kato H., Iwamoto A., Shih J.W., Alter H.J., Purcell R.H., Yoshikura H. (1993). Equilibrium centrifugation studies of hepatitis C virus: Evidence for circulating immune complexes. J. Virol..

[104] Lindenbach B.D., Evans M.J., Syder A.J., Wolk B., Tellinghuisen T.L., Liu C.C., Maruyama T., Hynes R.O., Burton D.R., McKeating J.A. (2005). Complete replication of hepatitis C virus in cell culture. Science.

[105] Wakita T., Pietschmann T., Kato T., Date T., Miyamoto M., Zhao Z., Murthy K., Habermann A., Krausslich H.G., Mizokami M. (2005). Production of infectious hepatitis C virus in tissue culture from a cloned viral genome. Nat. Med..

[106] Zhong J., Gastaminza P., Cheng G., Kapadia S., Kato T., Burton D.R., Wieland S.F., Uprichard S.L., Wakita T., Chisari F.V. (2005). Robust hepatitis C virus infection *in vitro*. Proc. Natl. Acad. Sci. USA.

[107] Gastaminza P., Kapadia S.B., Chisari F.V. (2006). Differential biophysical properties of infectious intracellular and secreted hepatitis C virus particles. J. Virol..

[108] Chang K.S., Jiang J., Cai Z., Luo G. (2007). Human apolipoprotein e is required for infectivity and production of hepatitis C virus in cell culture. J. Virol..

[109] Gastaminza P., Cheng G., Wieland S., Zhong J., Liao W., Chisari F.V. (2008). Cellular determinants of hepatitis C virus assembly, maturation, degradation, and secretion. J. Virol..

[110] Meex S.J., Andreo U., Sparks J.D., Fisher E.A. (2011). Huh-7 or HepG2 cells: Which is the better model for studying human apolipoprotein-B100 assembly and secretion?. J. Lipid. Res..

[111] Akazawa D., Date T., Morikawa K., Murayama A., Omi N., Takahashi H., Nakamura N., Ishii K., Suzuki T., Mizokami M. (2008). Characterization of infectious hepatitis C virus from liver-derived cell lines. Biochem. Biophys. Res. Commun..

[112] Gastaminza P., Dryden K.A., Boyd B., Wood M.R., Law M., Yeager M., Chisari F.V. (2010). Ultrastructural and biophysical characterization of hepatitis C virus particles produced in cell culture. J. Virol..

[113] Lindenbach B.D., Meuleman P., Ploss A., Vanwolleghem T., Syder A.J., McKeating J.A., Lanford R.E., Feinstone S.M., Major M.E., Leroux-Roels G. (2006). Cell culture-grown hepatitis C virus is infectious *in vivo* and can be recultured *in vitro*. Proc. Natl. Acad. Sci. USA.

[114] Podevin P., Carpentier A., Pene V., Aoudjehane L., Carriere M., Zaidi S., Hernandez C., Calle V., Meritet J.F., Scatton O. (2010). Production of infectious hepatitis C virus in primary cultures of human adult hepatocytes. Gastroenterology.

[115] Jammart B., Michelet M., Pecheur E.I., Parent R., Bartosch B., Zoulim F., Durantel D. (2013). VLDL-producing and HCV-replicating HepG2 cells secrete no more LVP than VLDL-deficient Huh7.5 cells. J. Virol..

[116] Aizaki H., Morikawa K., Fukasawa M., Hara H., Inoue Y., Tani H., Saito K., Nishijima M., Hanada K., Matsuura Y. (2008). Critical role of virion-associated cholesterol and sphingolipid in hepatitis C virus infection. J. Virol..

[117] Icard V., Diaz O., Scholtes C., Perrin-Cocon L., Ramiere C., Bartenschlager R., Penin F., Lotteau V., Andre P. (2009). Secretion of hepatitis C virus envelope glycoproteins depends on assembly of apolipoprotein B positive lipoproteins. PLoS One.

[118] Meunier J.C., Russell R.S., Engle R.E., Faulk K.N., Purcell R.H., Emerson S.U. (2008). Apolipoprotein c1 association with hepatitis C virus. J. Virol..

[119] Benga W.J., Krieger S.E., Dimitrova M., Zeisel M.B., Parnot M., Lupberger J., Hildt E., Luo G., McLauchlan J., Baumert T.F. (2010). Apolipoprotein E interacts with hepatitis C virus nonstructural protein 5A and determines assembly of infectious particles. Hepatology.

[120] Owen D.M., Huang H., Ye J., Gale M. (2009). Apolipoprotein E on hepatitis C virion facilitates infection through interaction with low-density lipoprotein receptor. Virology.

[121] Tomiyasu K., Walsh B.W., Ikewaki K., Judge H., Sacks F.M. (2001). Differential metabolism of human VLDL according to content of ApoE and ApoC-III. Arterioscler. Thromb. Vasc. Biol..

[122] Dreux M., Boson B., Ricard-Blum S., Molle J., Lavillette D., Bartosch B., Pecheur E.I., Cosset F.L. (2007). The exchangeable apolipoprotein ApoC-I promotes membrane fusion of hepatitis C virus. J. Biol. Chem..

[123] Mazumdar B., Banerjee A., Meyer K., Ray R. (2011). Hepatitis C virus E1 envelope glycoprotein interacts with apolipoproteins in facilitating entry into hepatocytes. Hepatology.

[124] Koutsoudakis G., Dragun J., Perez-Del-Pulgar S., Coto-Llerena M., Mensa L., Crespo G., Gonzalez P., Navasa M., Forns X. (2012). Interplay between basic residues of hepatitis C virus glycoprotein E2 with viral receptors, neutralizing antibodies and lipoproteins. PLoS One.

[125] Zhong J., Gastaminza P., Chung J., Stamataki Z., Isogawa M., Cheng G., McKeating J.A., Chisari F.V. (2006). Persistent hepatitis C virus infection *in vitro*: Coevolution of virus and host. J. Virol..

[126] Sabahi A., Marsh K.A., Dahari H., Corcoran P., Lamora J.M., Yu X., Garry R.F., Uprichard S.L. (2010). The rate of hepatitis C virus infection initiation *in vitro* is directly related to particle density. Virology.

[127] Agnello V., Abel G., Elfahal M., Knight G.B., Zhang Q.X. (1999). Hepatitis C virus and other flaviviridae viruses enter cells via low density lipoprotein receptor. Proc. Natl. Acad. Sci. USA.

[128] Albecka A., Belouzard S., Op de Beeck A., Descamps V., Goueslain L., Bertrand-Michel J., Terce F., Duverlie G., Rouille Y., Dubuisson J. (2012). Role of low-density lipoprotein receptor in the hepatitis C virus life cycle. Hepatology.

[129] Bishop J.R., Passos-Bueno M.R., Fong L., Stanford K.I., Gonzales J.C., Yeh E., Young S.G., Bensadoun A., Witztum J.L., Esko J.D. (2010). Deletion of the basement membrane heparan sulfate proteoglycan type XVIII collagen causes hypertriglyceridemia in mice and humans. PLoS One.

[130] Zheng C., Murdoch S.J., Brunzell J.D., Sacks F.M. (2006). Lipoprotein lipase bound to apolipoprotein B lipoproteins accelerates clearance of postprandial lipoproteins in humans. Arterioscler. Thromb. Vasc. Biol..

[131] Zeisel M.B., Felmlee D.J., Baumert T.F. (2013). Hepatitis C virus entry. Curr. Top. Microbiol. Immunol..

[132] Lupberger J., Zeisel M.B., Xiao F., Thumann C., Fofana I., Zona L., Davis C., Mee C.J., Turek M., Gorke S. (2011). EGFR and EphA2 are host factors for hepatitis C virus entry and possible targets for antiviral therapy. Nat. Med..

[133] Zona L., Lupberger J., Sidahmed-Adrar N., Thumann C., Harris H.J., Barnes A., Florentin J., Tawar R.G., Xiao F., Turek M. (2013). HRas signal transduction promotes Hepatitis C virus cell entry by triggering assembly of the host tetraspanin receptor complex. Cell Host Microbe.

[134] Fraser R., Dobbs B.R., Rogers G.W. (1995). Lipoproteins and the liver sieve: The role of the fenestrated sinusoidal endothelium in lipoprotein metabolism, Atherosclerosis, and cirrhosis. Hepatology.

[135] Ishibashi S., Perrey S., Chen Z., Osuga J., Shimada M., Ohashi K., Harada K., Yazaki Y., Yamada N. (1996). Role of the low density lipoprotein (LDL) receptor pathway in the metabolism of chylomicron remnants. A quantitative study in knockout mice lacking the LDL receptor, apolipoprotein E, or both. J. Biol. Chem..

[136] Rohlmann A., Gotthardt M., Hammer R.E., Herz J. (1998). Inducible inactivation of hepatic LRP gene by cre-mediated recombination confirms role of LRP in clearance of chylomicron remnants. J. Clin. Invest..

[137] Wilsie L.C., Orlando R.A. (2003). The low density lipoprotein receptor-related protein complexes with cell surface heparan sulfate proteoglycans to regulate proteoglycan-mediated lipoprotein catabolism. J. Biol. Chem..

[138] Chen K., Liu M.L., Schaffer L., Li M., Boden G., Wu X., Williams K.J. (2010). Type 2 diabetes in mice induces hepatic overexpression of sulfatase 2, A novel factor that suppresses uptake of remnant lipoproteins. Hepatology.

[139] Hassing H.C., Mooij H., Guo S., Monia B.P., Chen K., Kulik W., Dallinga-Thie G.M., Nieuwdorp M., Stroes E.S., Williams K.J. (2012). Inhibition of hepatic sulfatase-2 *in vivo*: A novel strategy to correct diabetic dyslipidemia. Hepatology.

[140] MacArthur J.M., Bishop J.R., Stanford K.I., Wang L., Bensadoun A., Witztum J.L., Esko J.D. (2007). Liver heparan sulfate proteoglycans mediate clearance of triglyceride-rich lipoproteins independently of LDL receptor family members. J. Clin. Invest..

[141] Stanford K.I., Bishop J.R., Foley E.M., Gonzales J.C., Niesman I.R., Witztum J.L., Esko J.D. (2009). Syndecan-1 is the primary heparan sulfate proteoglycan mediating hepatic clearance of triglyceride-rich lipoproteins in mice. J. Clin. Invest..

[142] Trigatti B.L., Krieger M., Rigotti A. (2003). Influence of the HDL receptor SR-BI on lipoprotein metabolism and atherosclerosis. Arterioscler. Thromb. Vasc. Biol..

[143] Morrone D., Weintraub W.S., Toth P.P., Hanson M.E., Lowe R.S., Lin J., Shah A.K., Tershakovec A.M. (2012). Lipid-altering efficacy of ezetimibe plus statin and statin monotherapy and identification of factors associated with treatment response: A pooled analysis of over 21,000 subjects from 27 clinical trials. Atherosclerosis.

[144] Thomssen R., Bonk S. (2002). Virolytic action of lipoprotein lipase on hepatitis C virus in human sera. Med. Microbiol. Immunol..

[145] Andreo U., Maillard P., Kalinina O., Walic M., Meurs E., Martinot M., Marcellin P., Budkowska A. (2007). Lipoprotein lipase mediates hepatitis C virus (HCV) cell entry and inhibits HCV infection. Cell Microbiol..

[146] Shimizu Y., Hishiki T., Sugiyama K., Ogawa K., Funami K., Kato A., Ohsaki Y., Fujimoto T., Takaku H., Shimotohno K. (2010). Lipoprotein lipase and hepatic triglyceride lipase reduce the infectivity of hepatitis C virus (HCV) through their catalytic activities on HCV-associated lipoproteins. Virology.

[147] Maillard P., Walic M., Meuleman P., Roohvand F., Huby T., Le Goff W., Leroux-Roels G., Pecheur E.I., Budkowska A. (2011). Lipoprotein lipase inhibits hepatitis C virus (HCV) infection by blocking virus cell entry. PLoS One.

[148] Sun H.Y., Lin C.C., Lee J.C., Wang S.W., Cheng P.N., Wu I.C., Chang T.T., Lai M.D., Shieh D.B., Young K.C. (2012). Very low-density lipoprotein/lipo-viro particles reverse lipoprotein lipase-mediated inhibition of hepatitis C virus infection via apolipoprotein C-III. Gut.

[149] Barth H., Schafer C., Adah M.I., Zhang F., Linhardt R.J., Toyoda H., Kinoshita-Toyoda A., Toida T., Van Kuppevelt T.H., Depla E. (2003). Cellular binding of hepatitis C virus envelope glycoprotein E2 requires cell surface heparan sulfate. J. Biol. Chem..

[150] Barth H., Schnober E.K., Zhang F., Linhardt R.J., Depla E., Boson B., Cosset F.L., Patel A.H., Blum H.E., Baumert T.F. (2006). Viral and cellular determinants of the hepatitis C virus envelope-heparan sulfate interaction. J. Virol..

[151] Jiang J., Cun W., Wu X., Shi Q., Tang H., Luo G. (2012). Hepatitis C virus attachment mediated by apolipoprotein E binding to cell surface heparan sulfate. J. Virol..

[152] Kapadia S.B., Barth H., Baumert T., McKeating J.A., Chisari F.V. (2007). Initiation of hepatitis C virus infection is dependent on cholesterol and cooperativity between CD81 and scavenger receptor B type I. J. Viro.l.

[153] Fuki I.V., Meyer M.E., Williams K.J. (2000). Transmembrane and cytoplasmic domains of syndecan mediate a multi-step endocytic pathway involving detergent-insoluble membrane rafts. Biochem. J..

[154] Scarselli E., Ansuini H., Cerino R., Roccasecca R.M., Acali S., Filocamo G., Traboni C., Nicosia A., Cortese R., Vitelli A. (2002). The human scavenger receptor class B type I is a novel candidate receptor for the hepatitis C virus. EMBO. J..

[155] Barth H., Cerino R., Arcuri M., Hoffmann M., Schurmann P., Adah M.I., Gissler B., Zhao X., Ghisetti V., Lavezzo B. (2005). Scavenger receptor class B type I and hepatitis C virus infection of primary tupaia hepatocytes. J. Virol..

[156] Barth H., Schnober E.K., Neumann-Haefelin C., Thumann C., Zeisel M.B., Diepolder H.M., Hu Z., Liang T.J., Blum H.E., Thimme R. (2008). Scavenger receptor class B is required for hepatitis C virus uptake and cross-presentation by human dendritic cells. J. Virol..

[157] Catanese M.T., Ansuini H., Graziani R., Huby T., Moreau M., Ball J.K., Paonessa G., Rice C.M., Cortese R., Vitelli A. (2010). Role of scavenger receptor class B type I in hepatitis C virus entry: Kinetics and molecular determinants. J. Virol..

[158] Dreux M., Dao Thi V.L., Fresquet J., Guerin M., Julia Z., Verney G., Durantel D., Zoulim F., Lavillette D., Cosset F.L. (2009). Receptor complementation and mutagenesis reveal SR-BI as an essential HCV entry factor and functionally imply its intra- and extra-cellular domains. PLoS Pathog..

[159] Dreux M., Pietschmann T., Granier C., Voisset C., Ricard-Blum S., Mangeot P.E., Keck Z., Foung S., Vu-Dac N., Dubuisson J. (2006). High density lipoprotein inhibits hepatitis C virus-neutralizing antibodies by stimulating cell entry via activation of the scavenger receptor BI. J. Biol. Chem..

[160] Haberstroh A., Schnober E.K., Zeisel M.B., Carolla P., Barth H., Blum H.E., Cosset F.L., Koutsoudakis G., Bartenschlager R., Union A. (2008). Neutralizing host responses in hepatitis C virus infection target viral entry at postbinding steps and membrane fusion. Gastroenterology.

[161] Zeisel M.B., Da Costa D., Baumert T.F. (2011). Opening the door for hepatitis C virus infection in genetically humanized mice. Hepatology.

[162] Zeisel M.B., Koutsoudakis G., Schnober E.K., Haberstroh A., Blum H.E., Cosset F.L., Wakita T., Jaeck D., Doffoel M., Royer C. (2007). Scavenger receptor class B type I is a key host factor for hepatitis C virus infection required for an entry step closely linked to CD81. Hepatology.

[163] Masson D., Koseki M., Ishibashi M., Larson C.J., Miller S.G., King B.D., Tall A.R. (2009). Increased HDL cholesterol and apoA-I in humans and mice treated with a novel SR-BI inhibitor. Arterioscler. Thromb. Vasc. Biol..

[164] Syder A.J., Lee H., Zeisel M.B., Grove J., Soulier E., Macdonald J., Chow S., Chang J., Baumert T.F., McKeating J.A. (2011). Small molecule scavenger receptor BI antagonists are potent HCV entry inhibitors. J. Hepatol..

[165] Out R., Hoekstra M., de Jager S.C., de Vos P., van der Westhuyzen D.R., Webb N.R., Van Eck M., Biessen E.A., Van Berkel T.J. (2005). Adenovirus-mediated hepatic overexpression of scavenger receptor class B type I accelerates chylomicron metabolism in C57BL/6J mice. J. Lipid Res..

[166] Van Eck M., Hoekstra M., Out R., Bos I.S., Kruijt J.K., Hildebrand R.B., Van Berkel T.J. (2008). Scavenger receptor BI facilitates the metabolism of VLDL lipoproteins *in vivo*. J. Lipid Res..

[167] Dao Thi V.L., Granier C., Zeisel M.B., Guerin M., Mancip J., Granio O., Penin F., Lavillette D., Bartenschlager R., Baumert T.F. (2012). Characterization of hepatitis C virus particle subpopulations reveals multiple usage of the scavenger receptor BI for entry steps. J. Biol. Chem..

[168] Zahid M.N., Turek M., Xiao F., Thi V.L., Guerin M., Fofana I., Bachellier P., Thompson J., Delang L., Neyts J. (2013). The post-binding activity of scavenger receptor BI mediates initiation of hepatitis C virus infection and viral dissemination. Hepatology.

[169] Sainz B., Barretto N., Martin D.N., Hiraga N., Imamura M., Hussain S., Marsh K.A., Yu X., Chayama K., Alrefai W.A. (2012). Identification of the Niemann-Pick C1-like 1 cholesterol absorption receptor as a new hepatitis C virus entry factor. Nat. Med..

[170] Haid S., Pietschmann T., Pecheur E.I. (2009). Low pH-dependent hepatitis C virus membrane fusion depends on E2 integrity, target lipid composition, and density of virus particles. J. Biol. Chem..

[171] Pecheur E.I., Diaz O., Molle J., Icard V., Bonnafous P., Lambert O., Andre P. (2010). Morphological characterization and fusion properties of triglyceride-rich lipoproteins obtained from cells transduced with hepatitis C virus glycoproteins. J. Biol. Chem..

[172] Voisset C., Lavie M., Helle F., Op De Beeck A., Bilheu A., Bertrand-Michel J., Terce F., Cocquerel L., Wychowski C., Vu-Dac N. (2008). Ceramide enrichment of the plasma membrane induces CD81 internalization and inhibits hepatitis C virus entry. Cell Microbiol..

[173] Rocha-Perugini V., Lavie M., Delgrange D., Canton J., Pillez A., Potel J., Lecoeur C., Rubinstein E., Dubuisson J., Wychowski C. (2009). The association of CD81 with tetraspanin-enriched microdomains is not essential for Hepatitis C virus entry. BMC Microbiol..

[174] Packard C.J., Shepherd J. (1997). Lipoprotein heterogeneity and apolipoprotein B metabolism. Arterioscler. Thromb. Vasc. Biol..

[175] Yamamoto M., Aizaki H., Fukasawa M., Teraoka T., Miyamura T., Wakita T., Suzuki T. (2011). Structural requirements of virion-associated cholesterol for infectivity, buoyant density and apolipoprotein association of hepatitis C virus. J. Gen. Virol..

[176] Lohmann V., Korner F., Koch J., Herian U., Theilmann L., Bartenschlager R. (1999). Replication of subgenomic hepatitis C virus RNAs in a hepatoma cell line. Science.

[177] Aizaki H., Lee K.J., Sung V.M., Ishiko H., Lai M.M. (2004). Characterization of the hepatitis C virus RNA replication complex associated with lipid rafts. Virology.

[178] Wang C., Gale M., Keller B.C., Huang H., Brown M.S., Goldstein J.L., Ye J. (2005). Identification of FBL2 as a geranylgeranylated cellular protein required for hepatitis C virus RNA replication. Mol. Cell.

[179] Kapadia S.B., Chisari F.V. (2005). Hepatitis C virus RNA replication is regulated by host geranylgeranylation and fatty acids. Proc. Natl. Acad. Sci. USA.

[180] Scholtes C., Diaz O., Icard V., Kaul A., Bartenschlager R., Lotteau V., Andre P. (2008). Enhancement of genotype 1 hepatitis C virus replication by bile acids through FXR. J. Hepatol..

[181] Chhatwal P., Bankwitz D., Gentzsch J., Frentzen A., Schult P., Lohmann V., Pietschmann T. (2012). Bile acids specifically increase hepatitis C virus RNA-replication. PLoS One.

[182] Romero-Brey I., Merz A., Chiramel A., Lee J.Y., Chlanda P., Haselman U., Santarella-Mellwig R., Habermann A., Hoppe S., Kallis S. (2012). Three-dimensional architecture and biogenesis of membrane structures associated with hepatitis C virus replication. PLoS Pathog..

[183] Herker E., Harris C., Hernandez C., Carpentier A., Kaehlcke K., Rosenberg A.R., Farese R.V., Ott M. (2010). Efficient hepatitis C virus particle formation requires diacylglycerol acyltransferase-1. Nat. Med..

[184] Ploen D., Hafirassou M.L., Himmelsbach K., Sauter D., Biniossek M.L., Weiss T.S., Baumert T.F., Schuster C., Hildt E. (2013). TIP47 plays a crucial role in the life cycle of hepatitis C virus. J. Hepatol..

[185] Miyanari Y., Atsuzawa K., Usuda N., Watashi K., Hishiki T., Zayas M., Bartenschlager R., Wakita T., Hijikata M., Shimotohno K. (2007). The lipid droplet is an important organelle for hepatitis C virus production. Nat. Cell Biol..

[186] Shimizu Y., Hishiki T., Ujino S., Sugiyama K., Funami K., Shimotohno K. (2011). Lipoprotein component associated with hepatitis C virus is essential for virus infectivity. Curr. Opin. Virol..

[187] Shi S.T., Lee K.J., Aizaki H., Hwang S.B., Lai M.M. (2003). Hepatitis C virus RNA replication occurs on a detergent-resistant membrane that cofractionates with caveolin-2. J. Virol..

[188] Hirata Y., Ikeda K., Sudoh M., Tokunaga Y., Suzuki A., Weng L., Ohta M., Tobita Y., Okano K., Ozeki K. (2012). Self-enhancement of hepatitis C virus replication by promotion of specific sphingolipid biosynthesis. PLoS Pathog..

[189] Sakamoto H., Okamoto K., Aoki M., Kato H., Katsume A., Ohta A., Tsukuda T., Shimma N., Aoki Y., Arisawa M. (2005). Host sphingolipid biosynthesis as a target for hepatitis C virus therapy. Nat. Chem. Biol..

[190] Weng L., Hirata Y., Arai M., Kohara M., Wakita T., Watashi K., Shimotohno K., He Y., Zhong J., Toyoda T. (2010). Sphingomyelin activates hepatitis C virus RNA polymerase in a genotype-specific manner. J. Virol..

[191] Reiss S., Rebhan I., Backes P., Romero-Brey I., Erfle H., Matula P., Kaderali L., Poenisch M., Blankenburg H., Hiet M.S. (2011). Recruitment and activation of a lipid kinase by hepatitis C virus NS5A is essential for integrity of the membranous replication compartment. Cell Host Microbe.

[192] Hsu S.H., Wang B., Kota J., Yu J., Costinean S., Kutay H., Yu L., Bai S., La Perle K., Chivukula R.R. (2012). Essential metabolic, anti-inflammatory, and anti-tumorigenic functions of miR-122 in liver. J. Clin. Invest..

[193] Tsai W.C., Hsu S.D., Hsu C.S., Lai T.C., Chen S.J., Shen R., Huang Y., Chen H.C., Lee C.H., Tsai T.F. (2012). MicroRNA-122 plays a critical role in liver homeostasis and hepatocarcinogenesis. J. Clin. Invest..

[194] Jopling C.L., Yi M., Lancaster A.M., Lemon S.M., Sarnow P. (2005). Modulation of hepatitis C virus RNA abundance by a liver-specific MicroRNA. Science.

[195] Shimakami T., Yamane D., Jangra R.K., Kempf B.J., Spaniel C., Barton D.J., Lemon S.M. (2012). Stabilization of hepatitis C virus RNA by an Ago2-miR-122 complex. Proc. Natl. Acad. Sci. USA.

[196] Sarasin-Filipowicz M., Krol J., Markiewicz I., Heim M.H., Filipowicz W. (2009). Decreased levels of microRNA miR-122 in individuals with hepatitis C responding poorly to interferon therapy. Nat. Med..

[197] Bulankina A.V., Deggerich A., Wenzel D., Mutenda K., Wittmann J.G., Rudolph M.G., Burger K.N., Honing S. (2009). TIP47 functions in the biogenesis of lipid droplets. J. Cell Biol..

[198] Martin S., Parton R.G. (2006). Lipid droplets: A unified view of a dynamic organelle. Nat. Rev. Mol. Cell Biol..

[199] Sato S., Fukasawa M., Yamakawa Y., Natsume T., Suzuki T., Shoji I., Aizaki H., Miyamura T., Nishijima M. (2006). Proteomic profiling of lipid droplet proteins in hepatoma cell lines expressing hepatitis C virus core protein. J. Biochem..

[200] Boulant S., Douglas M.W., Moody L., Budkowska A., Targett-Adams P., McLauchlan J. (2008). Hepatitis C virus core protein induces lipid droplet redistribution in a microtubule- and dynein-dependent manner. Traffic.

[201] Boulant S., Targett-Adams P., McLauchlan J. (2007). Disrupting the association of hepatitis C virus core protein with lipid droplets correlates with a loss in production of infectious virus. J. Gen. Virol..

[202] Shavinskaya A., Boulant S., Penin F., McLauchlan J., Bartenschlager R. (2007). The lipid droplet binding domain of hepatitis C virus core protein is a major determinant for efficient virus assembly. J. Biol. Chem..

[203] Targett-Adams P., Hope G., Boulant S., McLauchlan J. (2008). Maturation of hepatitis C virus core protein by signal peptide peptidase is required for virus production. J. Biol. Chem..

[204] Schuster C., Lefevre M., Baumert T.F. (2011). Triglyceride synthesis and hepatitis C virus production: Identification of a novel host factor as antiviral target. Hepatology.

[205] Kaul A., Stauffer S., Berger C., Pertel T., Schmitt J., Kallis S., Zayas M., Lohmann V., Luban J., Bartenschlager R. (2009). Essential role of cyclophilin A for hepatitis C virus replication and virus production and possible link to polyprotein cleavage kinetics. PLoS Pathog..

[206] Anderson L.J., Lin K., Compton T., Wiedmann B. (2011). Inhibition of cyclophilins alters lipid trafficking and blocks hepatitis C virus secretion. Virol. J..

[207] Olmstead A.D., Knecht W., Lazarov I., Dixit S.B., Jean F. (2012). Human subtilase SKI-1/S1P is a master regulator of the HCV Lifecycle and a potential host cell target for developing indirect-acting antiviral agents. PLoS Pathog..

[208] Huang H., Sun F., Owen D.M., Li W., Chen Y., Gale M., Ye J. (2007). Hepatitis C virus production by human hepatocytes dependent on assembly and secretion of very low-density lipoproteins. Proc. Natl. Acad. Sci. USA.

[209] Perlemuter G., Sabile A., Letteron P., Vona G., Topilco A., Chretien Y., Koike K., Pessayre D., Chapman J., Barba G. (2002). Hepatitis C virus core protein inhibits microsomal triglyceride transfer protein activity and very low density lipoprotein secretion: A model of viral-related steatosis. FASEB. J..

[210] Petit J.M., Benichou M., Duvillard L., Jooste V., Bour J.B., Minello A., Verges B., Brun J.M., Gambert P., Hillon P. (2003). Hepatitis C virus-associated hypobetalipoproteinemia is correlated with plasma viral load, steatosis, and liver fibrosis. Am. J. Gastroenterol..

[211] Jiang J., Luo G. (2009). Apolipoprotein E but not B is required for the formation of infectious hepatitis C virus particles. J. Virol..

[212] Steenbergen R.H., Joyce M.A., Lund G., Lewis J., Chen R., Barsby N., Douglas D., Zhu L.F., Tyrrell D.L., Kneteman N.M. (2010). Lipoprotein profiles in SCID/uPA mice transplanted with human hepatocytes become human-like and correlate with HCV infection success. Am. J. Physiol. Gastrointest. L..

[213] Coller K.E., Heaton N.S., Berger K.L., Cooper J.D., Saunders J.L., Randall G. (2012). Molecular determinants and dynamics of hepatitis C virus secretion. PLoS Pathog..

[214] Amako Y., Sarkeshik A., Hotta H., Yates J., Siddiqui A. (2009). Role of oxysterol binding protein in hepatitis C virus infection. J. Virol..

[215] Amako Y., Syed G.H., Siddiqui A. (2011). Protein kinase D negatively regulates hepatitis C virus secretion through phosphorylation of oxysterol-binding protein and ceramide transfer protein. J. Biol. Chem..

[216] Roe B., Kensicki E., Mohney R., Hall W.W. (2011). Metabolomic profile of hepatitis C virus-infected hepatocytes. PLoS One.

